# Trophic and temporal dynamics of macrophage biology in human inner ear organogenesis

**DOI:** 10.3389/fimmu.2025.1690583

**Published:** 2025-11-18

**Authors:** Yidi Deng, Boaz Ehiogu, Emilia Luca, Alain Dabdoub, Kim-Anh Lê Cao, Christine A. Wells, Bryony A. Nayagam

**Affiliations:** 1 Melbourne Integrative Genomics, School of Mathematics and Statistics, The University of Melbourne, Parkville, ACT, Australia; 2 Research School of Finance, Actuarial Studies & Statistics, The Australian National University, Canberra, ACT, Australia; 3 Sunnybrook Research Institute, Toronto, ON, Canada; 4 Department of Laboratory Medicine and Pathobiology, University of Toronto, Toronto, ON, Canada; 5 Department of Otolaryngology – Head & Neck Surgery, University of Toronto, Toronto, ON, Canada; 6 Department of Anatomy and Physiology, The University of Melbourne, Parkville, VIC, Australia; 7 Department of Audiology and Speech Pathology, The University of Melbourne, Carlton, VIC, Australia

**Keywords:** inner ear, macrophages, organogenesis, hearing, balance

## Abstract

Recent single-cell transcriptomic approaches are uncovering the breadth and depth of cell diversity within the mammalian inner ear. Macrophages, detected from fetal week 5 in the human inner ear, persist into adulthood and yet remain poorly understood in terms of their origin and function. Using self-generated and public scRNA-seq data, we identified seven distinct macrophage subtypes spanning fetal weeks 7.5 to 16.4 and adulthood. Each macrophage subtype is linked to specific developmental stages and displays a unique gene expression profile. These findings corroborate earlier histological evidence of resident and non-resident macrophages in both the developing and adult human cochlea. We also showed that the human inner ear is seeded by macrophages from both embryonic and more definitive sources, corroborating studies in mice. By analyzing ligand–receptor interactions, we highlight potential macrophage contributions to inner ear organogenesis. This research provides new insights into the diverse roles of human inner ear macrophages.

## Introduction

The normal development of the inner ear requires a sophisticated orchestration of specialized cell differentiation and integration, ultimately giving rise to the exquisite organs of hearing and balance. However, the timeline and dynamic nature of this developmental process remain only partially understood. Recent single-cell transcriptomic studies have improved our understanding of the molecular phenotypes of the mammalian inner ear through differential gene expression analyses in both mice (1, 2) and humans (3). While these studies have focused primarily on the inner ear hair cells and neurons, there are at least 17 other cell types present in the developing human inner ear (HIE), including large numbers of mesenchymal cells, supporting cells, and also macrophages (3). Each cell type likely performs a specific, but as yet largely uncharacterized, function in the formation of this elaborate organ.

Inner ear macrophages (IEMs) have gained increasing attention for their innate and adaptive immune roles in the normal (4), noise-damaged (5), and cochlear-implanted (6, 7) HIE. In mice, they have also been implicated in repairing the utricle (8) and in protecting cochlear afferent neurons (9). During HIE development, macrophages are first detected at fetal week 5, marked by the expression of *IBA1* and *CD45* (10), and they clearly populate the adult cochlea (4). Nevertheless, little is known about the origin of human cochlear macrophages or their functional contributions to inner ear organogenesis.

Beyond traditional immune surveillance, macrophages have well-established roles in development and tissue homeostasis (11, 12). They infiltrate numerous organs in the developing human (13), where they perform diverse and critical functions for normal organogenesis. For instance, in the brain, macrophages have been shown to regulate neurogenesis, synaptic pruning, and the clearance of apoptotic cells during brain development, thereby shaping intricate neural circuits fundamental to normal function (14). In the retina, macrophages help pattern and vascularize developing tissues, ultimately ensuring proper pupil morphology (15). Similarly, in the lung, macrophages assist in alveolar development and surfactant homeostasis, facilitating proper respiratory function (16). Given the complexity of inner ear patterning, fluid homeostasis, and vascularization, along with the observed early presence of macrophages during inner ear development, these multifunctional cells may be key to better understanding the intricate processes of normal auditory and vestibular organ formation.

**Figure 1:**
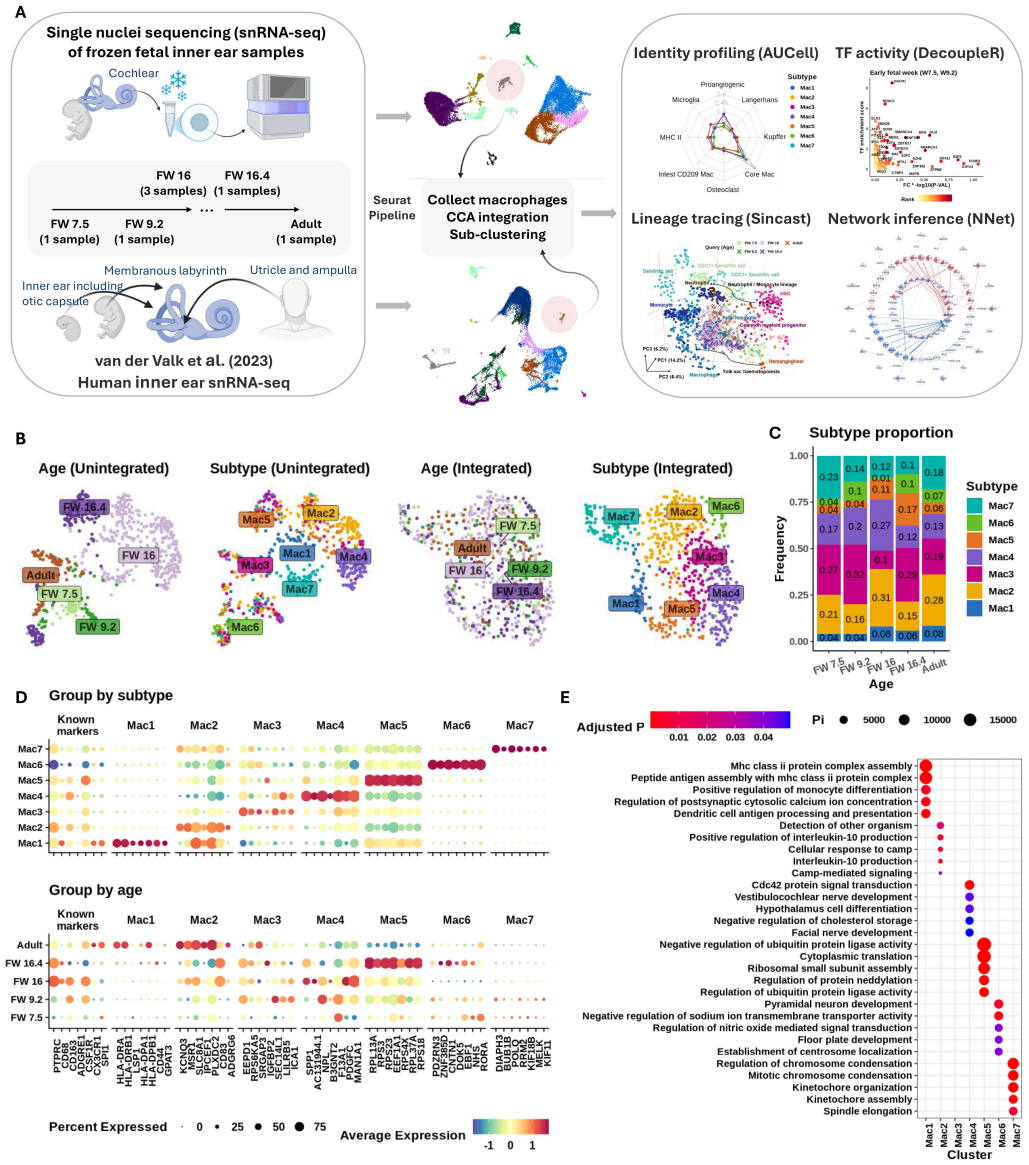
Seven fetal macrophage subtypes found in the developing inner ear. **(A)** Schematic of (L–R) tissue sample, integration, and analysis workflows used in this study. **(B)** Uniform manifold approximation and projection (UMAP) plots showing grouping of cells before (L) and after (R) Seurat integration. Macrophage subtypes were identified using Leiden clustering. **(C)** Stacked bar plot showing proportions (y-axis) of each subtype across developmental age (x-axis). **(D)** Expression of common macrophage markers and top-ranked discriminating genes (first seven genes) in each subtype. y-Axis shows categories of subtype (top) and age (bottom). Gene symbols shown on x-axis. Column headers indicate the subtypes that the markers represent. Expression shown on column-normalized z-score (red, highest expression; blue, lowest; yellow, mean expression value). The size of the dot indicates the percentage of the group expressing the gene of interest. **(E)** Gene set over-representation analysis of genes differentially expressed between macrophage subtypes. Subtypes shown on x-axis and gene ontology pathway terms on y-axis. Enrichment shown as circle size (relative proportion) and color (adjusted p-value).

In the present study, we offer the first comprehensive overview of macrophage molecular heterogeneity in the HIE (**Figure 1A**). By integrating both public and newly generated transcriptomic datasets, we assembled a comprehensive macrophage atlas covering key stages of HIE development: from early fetal weeks (FWs) 7.5 and 9.2, to middle FWs 16 and 16.4, and through to adulthood. We identified several distinct transcriptional profiles that define trophic roles for IEMs at different developmental ages and predict their likely modes of communication with other cell types. We also compared IEM phenotypes with macrophages present in other tissues during a similar window of human development (13). Collectively, the findings from this study provide essential insights into the diverse roles of IEMs during HIE development and pave the way for macrophage-targeted strategies to prevent or treat inner ear disorders.

## Results

### There are seven distinct macrophage subtypes present in the human inner ear

All data presented in this study were derived from single-nucleus RNA-sequencing data from the HIE.

The HIE was found to contain seven distinct macrophage subtypes (denoted as Mac 1 to Mac 7), each with a unique transcriptomic profile (**Figures 1B, D**). Each subtype was present at every timepoint examined (**Figure 1C**), despite differences in donor tissue, ages, and dissection processes. A summary of inner ear tissue descriptions at each age examined is provided in **Table 1**. Middle FW tissues yielded macrophages only from the cochlear modiolus rather than from the entire inner ear.

**Table 1:** Developmental ages of inner ear tissues used in this study.

Tissue age	Tissue collected	Equivalent Carnegie stage	Major developmental observations
FW 7.5	Whole inner ear (including the otic capsule)	Approx. Carnegie stage 18–19	Otic capsule pre-cartilaginous. Semi-circular ducts begin forming from thickened epithelial areas, and adjacent epithelial layers fuse. Cochlear duct is “L”-shaped.
FW 9.2	Whole inner ear (without the otic capsule)	Approx. Carnegie stage 23	Otic capsule cartilage is separated from the semicircular ducts by a pre-cartilaginous zone. Cochlea shows nearly 2½ turns. Labyrinth is near complete anatomical development. Ductus reuniens is well defined. Mesenchyme surrounding membranous labyrinth (otic capsule) chondrifies.
FW 16, 16.4	Cochlear modiolus (cochlea)	Fetal (post stage 23)	Adult-sized cochlear and vestibular organs. Functional vestibular system with cochlea approaching functional maturation. Capsule adjacent to membranous labyrinth undergoes vacuolization to form a cavity (perilymphatic space) around the membranous labyrinth, which fills with perilymph.
Adult	Utricle (vestibular system only)	Adult	Adult-sized, fully functional cochlear and vestibular organs. Healthy utricular tissue was removed and sequenced as part of a resection surgery.

This table summarizes the human inner ear tissues used in this study, including developmental ages, the collection, the corresponding Carnegie stages, and major morphological observations.

FW, fetal week.

Previous studies using immunohistochemistry to identify macrophages in the HIE have reported the expression of *PTPRC* (CD45), *CD68*, *CD163*, and *CX3CL1* (4, 10, 17). Consistently, we confirmed the expression of these markers (along with additional selected known macrophage markers *CSFR1* and *SPI1*) in both fetal and adult IEMs. However, these markers were non-discriminatory in defining any particular macrophage subtype (**Figure 1D** “known marker”). Note that we isolated the macrophage subset of the inner ear data based on the expression of *PTPRC* and *ITGAM* (CD11b), which are commonly used markers for gating macrophages (**Supplementary Figures 1A, B**). In contrast, *ADGRE1* (F4/80) expression was absent in our samples, in agreement with known species-specific expression patterns (18) and its enrichment in human eosinophils (19) (**Figure 1D**).

Differential gene expression (DE) analysis revealed distinct phenotypes for each macrophage subtype (**Figure 1D**; **Table 2**), suggesting unique functional roles in early development (Mac 6 and 7), trophic support, immune homeostasis (Mac 3, 4, and 5), and antigen presentation (Mac 1 and 2). The DE markers of Mac 5, 6, and 7 were distinctly expressed in their defining subtypes across all developmental stages, whereas those of Mac 1, 2, 3, and 4 formed distinct patterns mostly starting at FW 16 (**Supplementary Figure 2**). Further analysis showed that Mac 3 likely represents an intermediate cell state between Mac 2 and 4, given the relatively small number of differentially expressed genes (DEGs) that distinguish it (**Supplementary Figures 1C, D**).

**Table 2:** Summary of macrophage subtypes identified in the human inner ear (HIE).

Subtype	Representative DEGs	Enriched age(s)	Functional interpretation/GO pathways
Mac 1	*HLA-DRA*, *HLA-DPB1*, *CD74*, *CTSS*	Adult (some FWs 16 and 16.4)	Classical macrophage phenotype associated with antigen presentation and immune surveillance. Enriched for MHC-II complex assembly and sodium/calcium ion transport. Likely supports neural and hair cell homeostasis and contributes to endolymph ionic balance.
Mac 2	*MSR1*, *SLC8A1*, *KCNQ3*, *PLXCD2*, *ADGRG6*	Adult (some FWs 16 and 16.4)	Classical macrophage phenotype exhibiting active efferocytosis and regulation of ion efflux. Expresses growth factor-binding complexes and is enriched for pathways related to IL-10 production.
Mac 3	*IGFBP2*, *SEC14L1*, *EEPD1*, *ICA1*, *SRGAP3*	After FW 7.5	Transitional subtype between Mac 2 and Mac 4 showing trophic and metabolic support functions. Expresses genes involved in vascular and cochlear neurosensory development and preservation, lipid regulation, and actin remodeling.
Mac 4	*SPP1*, *F13A1*, *B3GNT2*	Mostly before FW 16.5	Specialized in tissue remodeling and repair. Expresses genes related to extracellular matrix crosslinking and modification and is enriched for pathways associated with neural development and *CDC42* signal transduction.
Mac 5	*IGFBP2*, *CSF1R*, ribosomal genes	Across all ages, mostly at FW 16.5	Trophic support macrophage with high ribosomal content. Aside from sharing key growth factor expression with Mac 3, Mac 5 exhibited the highest expression of *CSF1R*.
Mac 6	*CNTN1*, *DOK5*, *PDZRN3*, *RORA*	FW 16.5	Regulatory phenotype linked to neural differentiation. Engages Wnt and neuregulin signaling, suggesting involvement in sensory epithelial patterning and neuronal maturation.
Mac 7	*MELK*, *BUB1*, *KIF11*, *POLQ*, *RRM2*, *DIAPH3*	FWs 7.5 and 9.2	Highly proliferative macrophage subset enriched for genes controlling cell division, DNA synthesis and repair, and cytoskeletal remodeling. Reflects macrophage self-renewal and expansion during early otic development.

Each macrophage subtype (Mac 1 to Mac 7) is defined by unique transcriptional signatures (differentially expressed genes), developmental enrichment, and functional characteristics.

DEGs, differentially expressed genes; GO, gene ontology; FW, fetal week.

Specifically, Mac 7 populations were enriched for genes implicated in important roles in cell proliferation (*MELK*) (20), cell division (*BUB1* and *KIF11*) (21), DNA synthesis and repair (*POLQ* and *RRM2*) (22), and cytoskeletal remodeling (*DIAPH3*) (23, 24). Mac 6 was observed to have additional regulatory functions, expressing contactin 1 (*CNTN1*) (25), regulators of Wnt signaling (*DOK5* and *PDZRN3*) (26, 27), and the nuclear hormone receptor (*RORA*) (28), all of which have been implicated in cell proliferation events (**Figure 1E**). Notably, Wnt signaling plays a critical role in inner ear development and function (29–31). Further analysis of selected growth factor expression revealed that this macrophage subtype was involved in both neuregulin and Wnt5 signaling during early developmental ages (**Supplementary Figure 3**).

By contrast, Mac 3, 4, and 5 displayed broader associations with facial and vestibulocochlear nerve development (**Figure 1E**). For instance, they indicated a trophic phenotype through their relative expression of *IGFBP2* (Mac 3 and 5) (32), *PDGFC* (Mac 4) (33), and *CSF1R* (Mac 5) (34). These growth factors and receptors are vital for both vascular and cochlear neurosensory development and preservation. Additionally, Mac 4 showed functional specialization in tissue remodeling, evidenced by the expression of secreted phosphoprotein 1 (*SPP1*) (35), transglutaminase *F13A1* (36), and acetylglucosaminyltransferase *B3GNT2* (37), which are genes involved in crosslinking and modifying matrix proteins.

Mac 2 and 3 also expressed mediators of efferocytosis, including *MSR1* (a scavenger receptor), *SEC14L1* (a lipid co-factor that inhibits RIG-I signaling) (38), *EEPD1* (involved in cholesterol efflux) (39), *ICA1* (lipid complexing and receptor trafficking) (40), and *SRGAP3* (a regulator of actin dynamics via *RAC1*) (41). They also expressed regulators of calcium and potassium efflux (*KCNQ3* and *SLC8A1*) (42, 43), along with growth factor-binding complexes such as *IGFBP2*, *PLXCD2* (PEDF binding) (44), and the adhesion G-protein-coupled receptor *ADGRG6*. While Mac 2 markers were predominantly expressed in adult IEMs, Mac 3 markers were enriched in fetal IEMs around FW 16 (**Figure 1D**, bottom panel).

Mac 1 and 2 populations represented a mature, “classical” macrophage phenotype characterized by immune surveillance functions. Their putative roles were supported by the expression of numerous HLA transcripts (**Figure 1D**), indicating active antigen presentation and MHC-II regulation. Over-representation analysis of subtype markers further revealed enrichment in sodium and calcium ion transport pathways (**Figure 1E**), suggesting a possible role for Mac 1 and 2 in supporting hair cell and neural function. In addition, these macrophages may contribute to the maintenance of inner ear fluid (endolymph) homeostasis, given that these cations are critical for the generation of neural action potentials that underpin hearing and balance (45). Mac 1 also exhibited monocyte-like features and was most abundant from FW 16 through adulthood, comprising more than 6% of the population (**Figure 1C**).

### Age-dependent recruitment of macrophages to the inner ear shows defined subtypes present at distinct times

Having characterized seven macrophage subtypes via DE analysis, we next investigated whether specific macrophage phenotypes were present or absent at different stages of inner ear development. **Figures 1C, D** indicate a possible enrichment of Mac 1 and 2 in adult tissue; Mac 3, 4, and 5 at middle FWs; and Mac 6 and 7 at early FWs. To test this, we performed principal component analysis (PCA) on the expression of established macrophage module genes from Wang et al. (46) to examine their association with developmental age and IEM subtypes.

**Figure 2:**
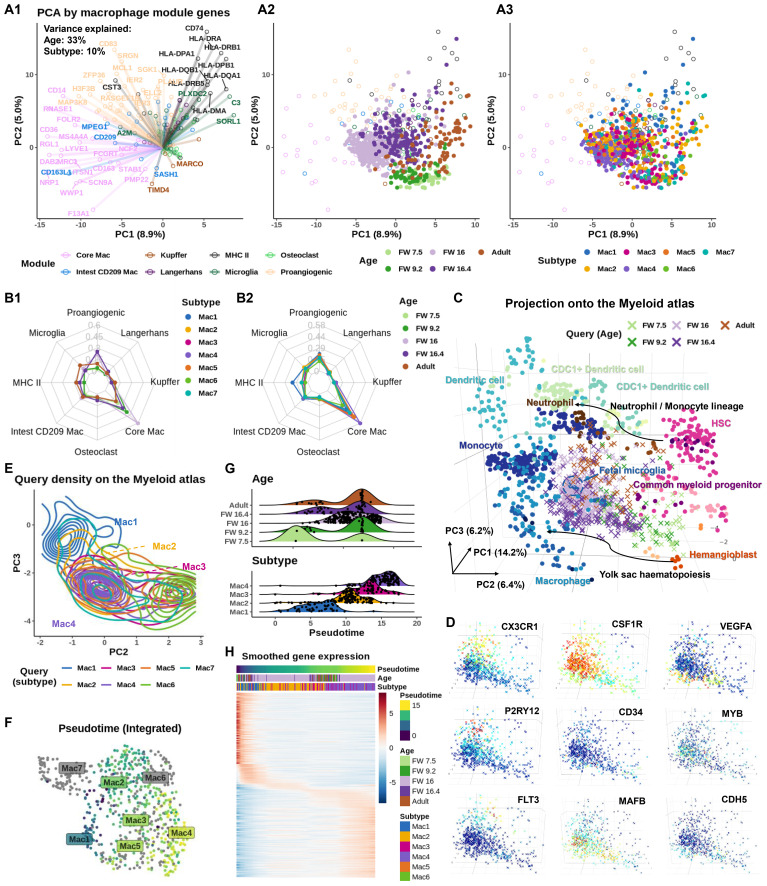
Age-dependent recruitment of macrophages to the inner ear shows defined subtypes present at distinct times. **(A)** Biplot showing principal component analysis (PCA) based on macrophage module genes (46). (A1) Circles represent gene loadings, colored by tissue module. PC scores of inner ear macrophages (IEMs) denoted as dots, colored by (A2) donor age and (A3) subtypes. **(B)** Radar graphs showing AUCell score of macrophage module genes at different (B1) developmental stages and (B2) different IEM subtypes in the inner ear **(C)** Projection of IEM subtypes to the Stemformatics.org myeloid atlas. Atlas samples denoted as circles and projected inner ear data as crosses. Inner ear samples colored by age. **(D)** Projected IEMs colored by the expression of CX3CR1 (adult), CSF1R (all), VEGFA [fetal weeks (FWs) 7.5, 9.2, and 16.4], P2RY12 (FW 16 and adult), CD34 (FWs 7.5 and 9.2), MYB and FLT3 (FW 16 and adult), and MAFB and CDH5 (FWs 7.5, 9.2, and 16.4). **(E)** Contour map of projected IEMs colored by subtypes demonstrates a continuum between Mac 1, 2, 3, and 4. **(F)** Uniform manifold approximation and projection (UMAP) plot, colored by pseudotime inferred using b mn, shot with Mac 1 selected as the root, highlights a trajectory of projected IEMs. Mac 5, 6, and 7 are excluded from the trajectory. **(G)** Pseudotime alignment of macrophages shows subtype (bottom plot), rather than age (top plot), as the strongest predictor of pseudotime on the Stemformatics atlas. **(H)** A heatmap of the top 500 genes correlated with pseudotime, clustered along the pseudotime axis, showing transition from Mac 1 to Mac 4.

The biplot in **Figure 2A** overlays i) a loading plot (**Figure 2A1**), which highlights the genes that drive variance, with ii) the projection of IEMs onto the PCA space (**Figures 2A2,A3**). The cells separate clearly by developmental ages, whereas subtype has little influence, suggesting that IEMs acquire distinct module identities during development. FW 16 IEMs displayed a strong core macrophage identity, while adult IEMs exhibited MHC-II and microglial signatures from the opposite side of the PCA. FW 16.4 macrophages occupied an intermediate, pro-angiogenic niche, and early FW IEMs clustered at the center of the PCA, indicating a relatively immature state. In additional studies, we showed that the population of IEMs that we have analyzed shares the greatest similarity with macrophage populations in the skin and brain during human development (**Supplementary Figure 4**).

We validated these findings using AUCell (47), which calculates module activity scores per cell in a manner that is robust to batch effects. This confirmed the age-dependent module activities (**Figure 2B1**). When examining module scores by subtypes, all subtypes exhibited a strong core macrophage identity. Notably, Mac 1 showed the highest co-adoption of MHC-II, microglial, and pro-angiogenic markers, consistent with a mature, efferocytotic, and antigen-presenting phenotype enriched in adult IEMs (**Figure 1E**).

To trace the age-dependent development of IEMs, we used Sincast (48) to benchmark their transcriptional identities against a myeloid atlas (49) consisting of bulk transcriptomic data from 44 independent studies. Note that pseudotime analysis cannot be meaningfully applied to compare developmental ages, which are confounded by tissue origin (**Supplementary Figures 5A–C** nevertheless show the inference). In contrast, reference-based identity profiling is less sensitive to batch variation across datasets. Sincast projected query IEMs onto the PCA space of the atlas, revealing two developmental trajectories: one from common myeloid progenitors (CMPs)/hematopoietic stem cells (HSCs) and the second from a hemangioblast-like progenitor (HLP), both of which ultimately differentiated into a fetal microglial-like or mature macrophage phenotype (**Figure 2C**) (50). We next examined the expression of key markers associated with macrophage differentiation (**Figure 2D**). Consistent with previous reports, the fractalkine receptor *CX3CR1*, a marker of long-lived tissue-resident macrophages (51), was most enriched in adult IEMs. Conversely, *CD34*, a common marker for HSCs and endothelial progenitors (52), was mainly expressed by the early FW IEMs. *VEGFA* expression peaked in the middle FW populations when the network of cochlear vasculature is increasing in density. The purinergic receptor *P2RY12*, implicated in regulating microglial surveillance and cAMP signaling (53), was enriched in both middle FW and adult IEMs. As expected, *CSF1R*, which is essential for macrophage survival and homeostasis, was broadly expressed across all developmental stages. Interestingly, *FLT3* and *MYB*, key regulators of early hematopoiesis in bone marrow (54, 55), were exclusively expressed in IEMs aligned with the CMP/HSC trajectory. In contrast, *CDH5* (VE-cadherin), an endothelial marker suggestive of a hemogenic endothelium origin (56), was expressed along the HLP trajectory. These IEM trajectory analyses were further interrogated using the Bian et al., (13) dataset and support a dual contribution to IEM seeding that is consistent with other organs (**Supplementary Figures 5D, E**). Together, these results reveal distinct developmental pathways for macrophage ontogeny in the inner ear.

Independent of the major age-dependent trajectories shown in **Figure 2C**, we observed an additional continuous spectrum of macrophage phenotypes spanning Mac 1, 2, 3, and 4 in order along the myeloid atlas (**Figure 2E**). Unlike developmental ages, IEM subtypes are not confounded by tissue origin, allowing this spectrum to be traced by pseudotime analysis. Slingshot (57), applied to the projected IEMs with Mac 1 as the root, confirmed this spectrum by revealing a trajectory extending from Mac 1 to Mac 4, while automatically excluding Mac 6, Mac 7, and most of Mac 5 macrophages due to their lack of connectivity in the inferred lineage graph (**Figure 2F**). When pseudotime was stratified by developmental age and subtype (**Figure 2G**), it showed no correlation with age but aligned strongly with subtype, indicating that this trajectory reflects a phenotypic transition independent of age-related differentiation. To further support this continuum, we examined smoothed gene expression patterns across macrophage subtypes (**Figure 2H**). The results revealed a gradual shift in gene expression from Mac 1 to 4, with Mac 2 and 3 sharing similar profiles and representing intermediate states.

### Inner ear macrophages alternate their gene regulation profile during early development

**Figure 3:**
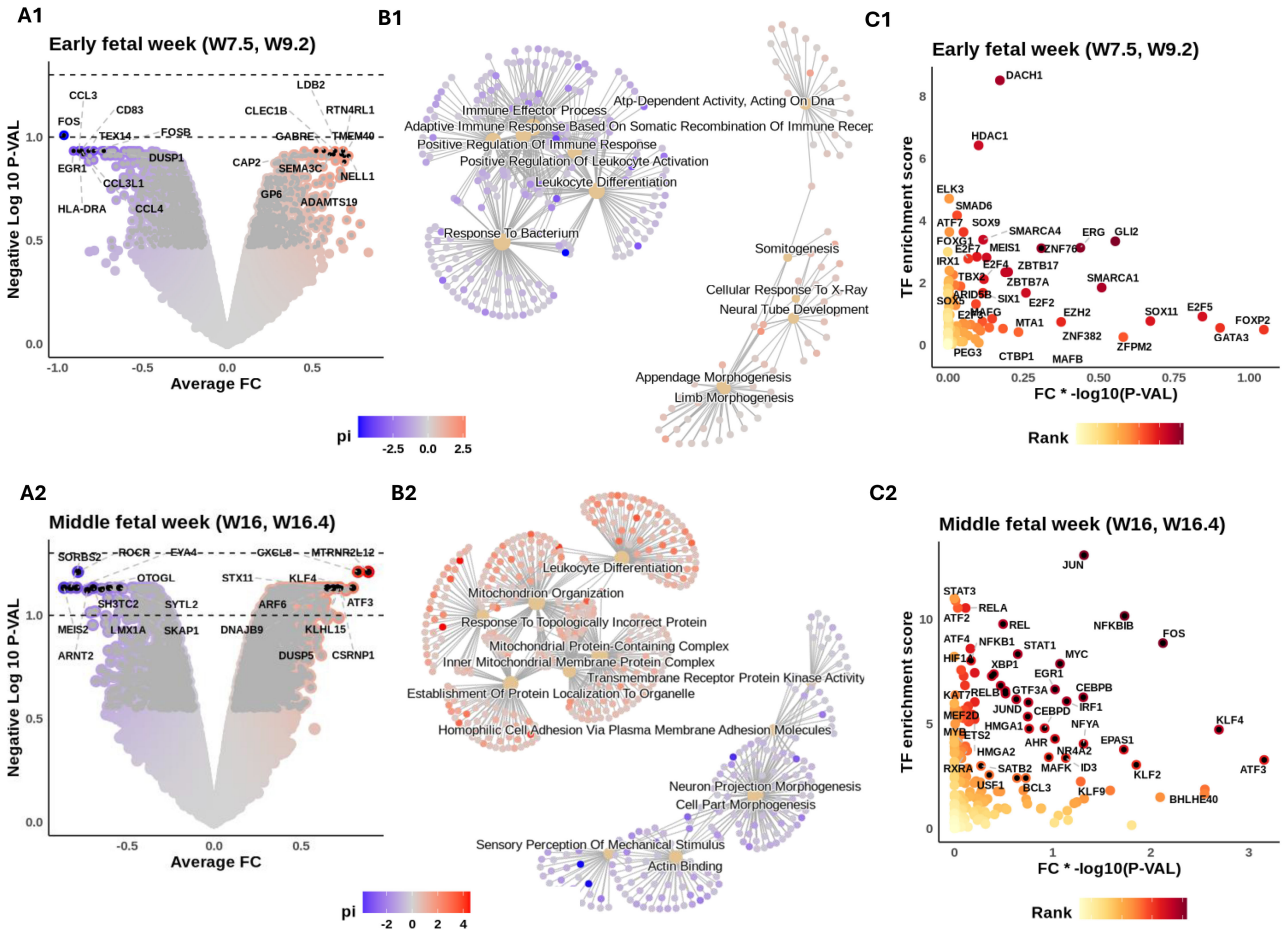
Intra- and intercellular signaling reveal macrophage functional heterogeneity during fetal development. Inner ear macrophages (IEMs) within each sample are aggregated into a pseudobulk sample. Differential expression was conducted to compare fetal week (FW) 7.5 and 9.2 samples to later developmental stages (middle FWs 16 and 16.4 and the adult). Similarly, middle FW samples are also compared to the other two age groups. The results for the early and middle FWs are shown in subpanels numerated 1 and 2, respectively. **(A)** Volcano illustrating the differential gene expression (DE) results. Genes colored according to their π-value = −log10(p-value) * fold change. Symbols of top 10 up- and downregulated genes are shown. Dashed lines show adjusted p-value thresholds equal to 0.1 (lower) and 0.05 (upper). **(B)** Gene set enrichment analysis of age markers. The top 6 enriched terms for up- and downregulated genes are shown. Nodes connecting the terms are associated markers, colored by their π-values. **(C)** DecoupleR transcription factor (TF) activity inference on DE results. x-Axis: π-values of TFs. y-Axis: DecoupleR activity scores of TFs. TFs colored by their multiplied ranking in π-values and activity scores. Symbols of top 50 ranked TFs are shown.

Having investigated the influence of developmental age on macrophage subtypes, we next examined genes that were significantly up- or downregulated in early FWs 7.5 and 9.2 compared to later developmental stages (middle FWs 16 and 16.4 and adult; **Figure 3A1**). A summary of results is presented in **Table 3**.

**Table 3:** Summary of key transcriptional regulators and signaling pathways identified in inner ear macrophages (IEMs) across developmental stages.

Stage	Key transcription factors	Signaling axes and functional highlights
Early FW 7.5	*SMAD3*, *SMAD6* (regulator of TGF-β signaling); *DACH1* (stria vascularis development), *FOXG1* (neural development); *HDAC1* (cell proliferation and migration); *MEIS1*, *ERG* (embryonic hematopoiesis); *GLI2* (Hedgehog signaling).	*TGFBR2–SMAD3* and *FGFR1–CREBBP* axes promote expression of P-cadherin (*CDH3*), suggesting macrophage involvement in maintaining epithelial integrity and formation of the tunnel of Corti. Early IEMs support structural maturation via regulation of cell–cell adhesion and tissue organization.
Early FW 9.2	*SOX5*, *SOX6* (cartilage formation).	Activation of *SOX5/6*-mediated transcription regulating chondro-osteogenic and neurogenic targets such as *NELL1*, implying roles in otic capsule ossification and neural pathfinding within the developing cochlea. Macrophage-specific utilization of developmental SOX signaling.
Middle FWs 16 and 16.4	*NFKBIB*, *RELA*, *RELB*, *NFKB2*, *NKRF* (NF-κB family members); *ATF4*, *IRF1*, *TBP*, *XBP1* (pro-inflammatory); *STAT3*, *MYC*, *EGR1*, *FOS*, *HIF1A* (reparative or metabolic regulators)	Macrophage subsets that exhibit distinct immune-metabolic programs are present: Mac 2: *NFKB1*-mediated signaling relaying from *IL1RAP*, *TNFRSF1A*, *CCR1*, and *TLR4* to FOS family genes, indicating transition activation. Mac 4: *IRF1*-driven pathway downstream of *TNFRSF1B*, *IL6ST*, *IL10RA* regulating *CDKN1A*, and *SOCS3*, thus promoting phagocytosis. Both subtypes engage a *NOTCH1–HIF1A* axis responding to *VEGFA*-induced hypoxia during vascular ingrowth. Indicates metabolic reprogramming and immune adaptation before hearing onset.

NeighbourNet and DecoupleR analyses highlight stage-specific transcription factors (TFs) and receptor–TF signaling axes underlying macrophage regulatory dynamics.

FWs, fetal weeks.

Early FW IEMs showed enriched expression of *RTN4RL1* and *SEMA3C* (involved in the regulation of axonal outgrowth) (58), *GABRE* (critical for GABA-_A_ receptor production) (59), and *NELL1* (implicated in osteoclast differentiation and bone formation) (60). Subsequent gene set enrichment analysis (GSEA) of these age markers (**Figure 3B1**) revealed the potential involvement of IEMs in synaptic membrane development and limb morphogenesis, suggesting possible broader roles in neural, skeletal, and tissue differentiation during early development. To identify transcriptional regulators of these age markers, we applied DecoupleR for transcription factor (TF) activity inference (61). This analysis highlighted candidate regulators including *MEIS1* and *ERG* (essential regulators of embryonic hematopoiesis) (62, 63), *GLI2* (involved in Hedgehog signaling) (64), *SMAD6* (a negative regulator of TGF-β signaling) (65), *DACH1* and *FOXG1* (both important for nervous system development) (66, 67), and *HDAC1* (involved in controlling cell proliferation and migration) (68) (**Figure 3C1**).

We then repeated these analyses for middle FWs 16 and 16.4, with comparisons made to all other donor ages (i.e., early FWs and adult; **Figure 3A2**). Middle FW IEMs showed enriched expression of a broad range of immune-modulator genes, including *CXCL8* (inflammatory chemokines) (69), *STX11* (regulating vesicle exocytosis) (70), and *ARF6* (implicated in phagocytosis) (71) (**Figure 3A2**). The enrichment of *KLF4* is intriguing, given its possible role in controlling tissue macrophage identity (72). GSEA further highlighted the association of IEMs with inflammatory responses (**Figure 3B2**). The enrichment of mitochondrial activity suggests a metabolic switch at around FW 16, indicating a putative change in tissue microenvironment that occurs prior to the onset of hearing (**Figure 3B2**; **Table 3**). Subsequent TF activity inference revealed a large number of immune-related TFs likely responsible for driving these transcriptional shifts from early to middle FWs. These TFs include regulators of innate immunity from the NF-κB family (*NFKBIB*, *RELA*, *RELB*, *NFKB2*, and *NKRF*), key regulators of macrophage function, including both pro-inflammatory (*ATF4*, *IRF1*, and *XBP1*) (73, 74) and reparative (*STAT3*, *MYC*, *ERG1*, and *FOS*) (75–77) roles (**Figure 3C2**). In addition, *HIF1A* was highly enriched, providing further evidence for IEMs’ metabolic reprogramming around middle FWs.

### Inner ear macrophages adopt distinct molecular identities in response to the dynamic tissue environment during fetal development

We applied NeighbourNet analysis to reconstruct gene regulatory networks (GRNs) of IEMs across developmental stages, characterizing the dynamics of overarching regulatory patterns (78). This analysis prioritized the most significantly upregulated genes in each age group (identified by the DE analysis in **Figure 3**), predicted their regulatory interactions with TFs, and inferred upstream signaling cascades, starting from receptors that potentially transduce extracellular signals to regulate age marker expression through the predicted TF interactions. Complementing this, we employed NicheNet analysis (79) (**Supplementary Results 9.1**) to predict ligand sources, thereby tracing signaling origins and intercellular communication during HIE development. A summary of the results is presented in **Table 3**.

**Figure 4:**
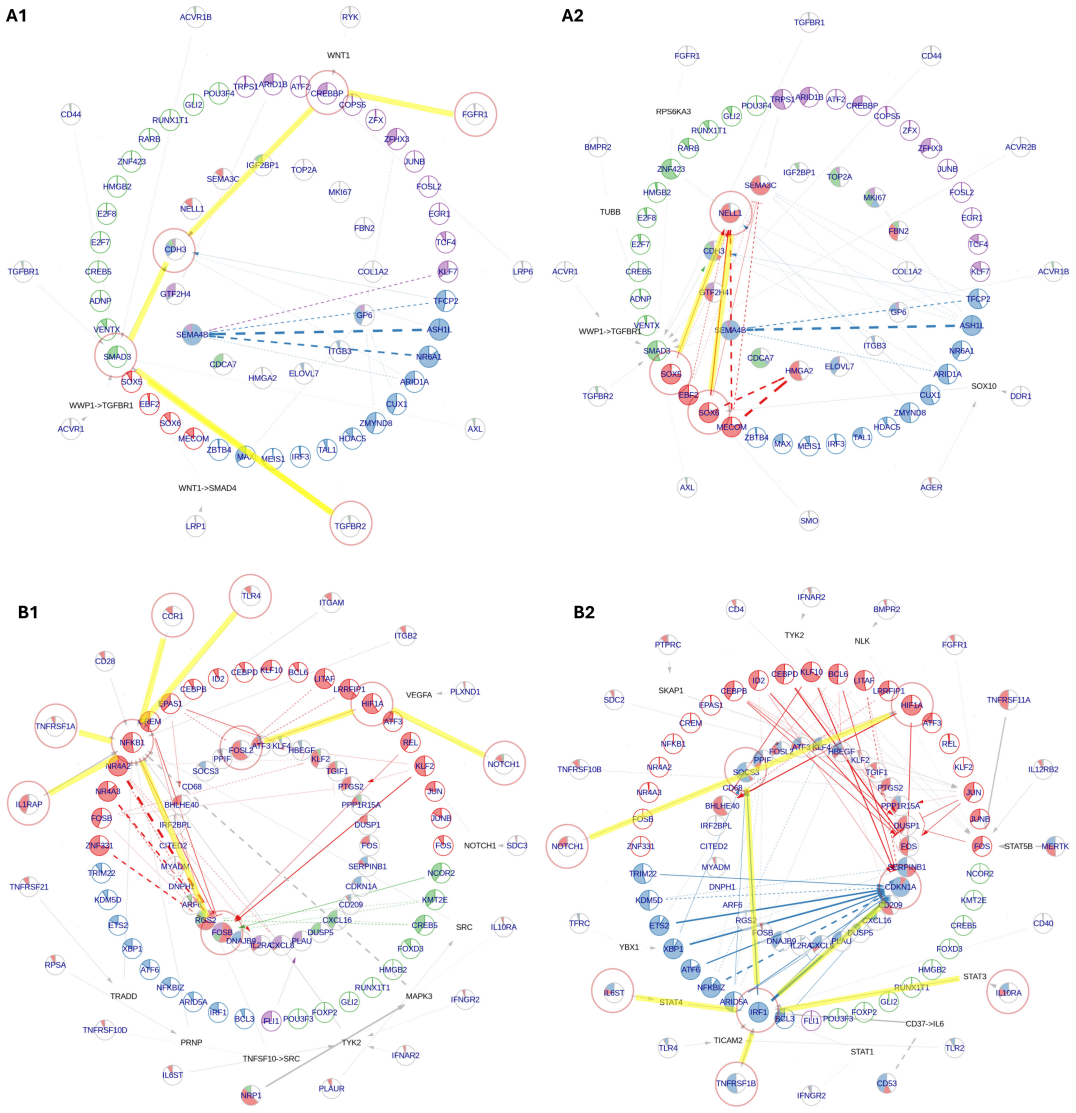
NeighbourNet inference of gene regulatory networks (GRNs) associated with the top 50 upregulated genes by early and middle fetal week (FW) inner ear macrophages (IEMs). The age markers are derived from differential gene expression analysis in **Figure 3A**. **(A)** GRNs for early FW markers, constructed primarily by IEMs at (A1) FW 7.5 and (A2) FW 9.2. **(B)** GRNs for middle FW markers, constructed primarily by (B1) Mac 2 and (B2) Mac 4 clusters. The macrophages used for GRN inference were automatically selected by the NeighbourNet algorithm as those exhibiting the most representative regulatory patterns. In each network, the innermost layer contains age markers, surrounded by their highly co-expressed transcription factors (TFs). Receptors are placed in the outermost layer, each connected to a TF predicted to mediate its regulatory influence. When the receptor–TF link is indirect, an additional layer displays the shortest inferred signaling path. Arrowheads indicate activation, bar-heads indicate repression, and dashed lines denote links with significant co-expression but not supported by prior knowledge or regulatory evidence. The color of each TF indicates its cluster, representing groups of TFs with similar co-expression patterns. The color of each target reflects the proportion of regulatory edges it receives from different TF clusters. The filling proportion of each pie represents the gene’s activation status: the more filled the pie, the higher the activity.

At FW 7.5, the GRN prominently highlighted the upregulation of P-cadherin (*CDH3*), driven by *TGFBR2*–*SMAD3* and *FGFR1*–*CREBBP* signaling axes (**Figure 4A1**). This suggests a potential role for early fetal IEMs in maintaining sensory epithelial integrity and facilitating the proper formation of the tunnel of Corti via the regulation of cell–cell adhesion (80). By FW 9.2, macrophages exhibited enriched SOX-family-mediated signaling involving *SOX5* and *SOX6*, which are TFs primarily known for their critical roles in chondrocyte and neuron differentiation (**Figure 4A2**) (81). In our IEMs, these TFs were predicted to regulate chondro-osteogenic (*NELL1*) and neurogenic (*NELL1* and *SEMA3C*) targets. Hence, this observation suggests a macrophage-specific utilization of *SOX5/6* to support HIE development by coordinating otic-capsule ossification and guiding cochlear neural pathfinding.

The signaling landscape significantly shifted in macrophages during the middle FWs, adopting classical macrophage signaling pathways involved in immune and tissue modulation. Interestingly, major signaling variations within these macrophages were primarily driven by the macrophage subtypes Mac 2 and Mac 4. Mac 2 displayed *NFKB1*-mediated pro-inflammatory signaling, relaying signals from IL1R (*IL1RAP*), TNFR1 (*TNFRSF1A*), CCR1, and TLR4 to upregulate FOS family genes, indicative of a transitional activation state (**Figure 4B1**) (82). In contrast, Mac 4 exhibited reduced NF-κB signaling, instead activating *IRF1*-mediated pathways downstream of TNFR2 (*TNFRSF1B*), IL6R (*IL6ST*), and IL10R (*IL10RA*) to regulate key phagocytosis mediators *CDKN1A* and *SOCS3* (**Figure 4B2**) (83, 84). Furthermore, both macrophage subtypes demonstrated the activation of a *NOTCH1*–*HIF1A* axis associated with macrophage responses to hypoxia and inflammation (85). Notably, NicheNet analysis predicted that middle FW IEMs were receiving pro-angiogenesis signals, *VEGFA* (vascular endothelial growth factor A) (**Supplementary Figure 6B**). The activation of the *NOTCH*–*HIF1A* axis can, therefore, be explained by macrophage adaptation to transient hypoxic tissue niches created during vascular ingrowth in middle FWs, when local metabolic demand briefly outpaces the new circulation (86). Collectively, our findings reveal a broad spectrum of macrophage identities and possible functional roles shaped by the complex and dynamic microenvironment in the developing HIE.

## Discussion

Recent single-cell studies have highlighted the heterogeneity of IEMs in mice, revealing distinct subtypes that may contribute to the intricate developmental processes (87). Our data build upon these studies and histological investigations by Steinacher et al. (10), who reported the presence of both resident and non-resident macrophages in the developing HIE between fetal weeks 7 and 15.

Using a transcriptional approach, we identified seven transcriptionally distinct IEM subtypes present over a similar window of HIE development (**Figure 1D**), with each subtype closely linked to a specific developmental age. For instance, we identified a proliferative population of macrophages that infiltrate the early developing HIE (Mac 7), followed by the emergence of subtypes associated with neural development (Mac 3 to 6), including ion homeostasis (Mac 6), and subsequently subtypes with characteristics of mature antigen-presenting macrophages (Mac 1 and 2). Despite differences in donor tissue age, dissection, and samples collected, we detected all seven macrophage subtypes at every timepoint examined. Together, these results highlight, for the first time, the breadth of macrophage phenotypes present in the early developing HIE and their multidisciplinary contributions to normal development.

Lineage tracing studies in mice have also provided insights into the developmental origins of IEMs, revealing their potential derivation from distinct embryonic sources (34, 87). Our data support these observations in human development, identifying inner ear seeding by both embryonic (yolk sac-derived) and more definitive (bone marrow-derived) macrophages during organogenesis. Specifically, we illustrated that FW 7.5 to 9.2 IEMs cluster most closely with multipotent hemangioblasts (yolk sac progenitors), whereas FW 16 and adult IEMs show a hematopoietic stem cell origin based on the projection to the myeloid atlas (**Figure 2C**). These analyses are supported by additional projections of extensive human macrophage datasets (13) obtained across organs in human development (**Supplementary Figure 4A–C**, **5D,E**). Moreover, presumptive yolk sac-derived IEMs (Mac 1 to 4) exhibit a distinct transitional trajectory that excludes Mac 5, 6, and 7 (**Figure 2E**). These data again support multiple ontogenies of IEMs. When projected onto a human fetal macrophage atlas (13), IEMs display a unique tissue identity, aligning most closely with developing macrophages found in the skin and the brain (**Supplementary Figures 4D–F**). Our analyses support the conclusions from Bian et al. (13) that diverse macrophage subtypes are found at defined anatomical sites during human development. The precise contribution of each of these distinct macrophage lineages to the heterogeneity and functional diversity in the HIE is open for future exploration.

Our transcriptomic approach not only illuminates key developmental processes but also reveals new signaling interactions, with direct applications to macrophage-associated hearing pathologies (88, 89). Our data implicate numerous well-characterized trophic signaling pathways of macrophages, including TGF-β, FGF, and semaphorin-neuropilin families in early developmental (FWs 7.5 and 9.2; **Figure 4A**, **Supplementary Figure 6A**), as well as *VEGFA*, *TNFSF*, *IGSF*, and *CNTN2* during the middle developmental stages (FWs 16 and 16.4; **Figure 4B**, **Supplementary Figure 6**). In particular, the *TGFB2* and *VEGF* pathways are predicted to regulate *SEMA3A/C* expression in early fetal cochlear macrophages (**Supplementary Figure 6A**). Although the ligand *SEMA3A*, known to be important for normal cochlear morphology and function (90), has traditionally been attributed to cochlear neurons and supporting cells (91), our analyses reveal that macrophages may be an additional cellular source.

We also identified a possible macrophage contribution to the newly discovered GABA signaling in the mammalian cochlea (92) through the GABA_A_ receptor subunit *GABRE* expression in early development (**Supplementary Figure 6B**). Further support for macrophage involvement in cochlear development is indicated by the enriched expression of *PDZRN3* in the Mac 6 population (**Figure 1D**), highlighting the potential role of this subtype in regulating Wnt signaling. Examining Wnt expression supports this hypothesis, illustrating Mac 6 as a source of Wnt5 ligands in HIE development (**Supplementary Figure 7**). Wnt signaling is critical for normal inner ear development (31), including *WNT5A* in correct hair cell function via planar cell polarity signaling (29, 93–95). In addition, neuregulin signaling has been shown to be important for neural survival in the mammalian cochlea (96), and our analyses indicate Mac 6 as a source of both *NRG1* and *NRG3* in HIE development (**Supplementary Figure 3**).

Macrophages are also expected to contribute to the establishment of the intricate cochlear vasculature. The predicted high TF activity of *DACH1* in early FW IEMs supports a critical role for macrophages in stria vascularis development (**Figure 3C**) (97). It is also possible that *DACH1* expression is under-represented in the middle FW and adult data, given that the stria vascularis is missing from these tissue dissections. *DACH1* is important for the development of endocochlear potential, with the knockdown of this TF causing hearing loss (98). Macrophages are therefore likely to play a pivotal role in normal cochlear development by orchestrating vascular formation, maintaining fluid homeostasis, and ultimately supporting the proper establishment of tonotopicity. These findings underscore the power of transcriptomic analyses in illuminating normal developmental processes, supporting the notion that IEMs play multidimensional roles far beyond their traditional immune functions. A deeper understanding of these diverse functions will not only enrich our fundamental knowledge of cochlear biology but also accelerate novel therapeutic strategies targeting immune-related, congenital, and age-related hearing loss (88, 89).

### Limitations

A challenge in the present study is the limited availability of human donor tissue. While we have captured early, middle, and late timepoints, we acknowledge that our conclusions are restricted to the tissues available. Therefore, there may be additional specialized macrophages missing from our analysis with roles in the developing inner ear, such as the bone-remodeling osteoclast. It is not known when osteoclasts become important in the human otic capsule—certainly, at week 7, this structure is more cartilaginous than bone (99). By week 9, we may expect some ossification of the otic capsule, but this was removed before profiling. The detection of multinucleated cells like osteoclasts is challenging with traditional droplet-based approaches, but future work, including *in situ* or spatial profiling, could help us reveal these additional macrophage subtypes.

Additionally, the paucity of human tissue has contributed to an imbalanced study design, in which macrophages from different age groups were collected from separate studies and tissues dissected slightly differently (as noted). We acknowledge that frozen samples can yield variability in tissue quality. Consequently, comparisons among age groups may be confounded by batch effects and may not fully capture the variability present at each developmental stage. While we recognize that the multiple ontologies presented in **Figure 2C** would ideally be followed by proper pseudotime inference and *de novo* marker identification along pseudotime, the substantial technical differences between our self-sequenced data and the dataset from van der Valk et al. (3) preclude such integrative analyses.

## Materials and methods

The key resources used in this study, including the datasets analyzed and the software employed for the analysis, are summarized in **Table 4**.

**Table 4:** Summary of the key resources used in this study, including the datasets analyzed and the software employed.

Resource name	Source	Identifier
Data		
snRNA-seq human inner ear Fetal weeks 16, 16.4	Self-generated	https://zenodo.org/records/15328483/
snRNA-seq human inner ear Fetal weeks 7.5, 9.2, and adult	van der Valk et al. (3)	GEO: GSE213796
Myeloid cell atlas	Stemformatics and Rajab et al. (49)	https://www.stemformatics.org/
Macrophage module genes	Wang et al. (46)	10.1016/j.cell.2023.08.019
Software		
R v4.2.1	The R Project	https://www.r-project.org/
Seurat v5.0.3	Hao et al. (101)	https://satijalab.org/
scDblFinder v1.17.2	Germain et al. (109)	https://github.com/plger/scDblFinder
ggplot2 v3.5.0	Wickham (110)	https://ggplot2.tidyverse.org/
clusterProfiler v4.11.1	Yu et al. (104)	https://bioconductor.org/packages/clusterProfiler/
AUCell v1.18.1	Aibar et al. (47)	https://bioconductor.org/packages/AUCell/
Sincast v1.0.0	Deng et al. (48)	https://github.com/meiosis97/Sincast
Slingshot v2.7.0	Street et al. (57)	https://bioconductor.org/packages/slingshot/
limma v3.52.4	Ritchie et al. (106)	https://bioconductor.org/packages/limma/
decoupleR v2.9.7	Badia-i Mompel et al. (61)	https://bioconductor.org/packages/decoupleR/
nichenetr v2.2.0	Browaeys et al. (79)	https://github.com/saeyslab/nichenetr/

Resource name indicates the name of the resource, Source refers to the publication or project from which the resource was generated, and Identifier provides the hyperlink for accessing the resource.

snRNA-seq, single-nucleus RNA-sequencing.

### Ethics approval

De-identified human fetal samples were obtained from the Research Centre for Women’s and Infants’ Health (RCWIH) BioBank with approval from the Research Ethics Boards of Mount Sinai Hospital (ID# 20-0003-E) and Sunnybrook Health Sciences Centre (Project Identification Number 1514).

No compensation was provided for participation in this study.

### Human spiral ganglion collection and dissection

**Table 1** summarizes the human inner ear tissues used in this study, including their developmental ages, collection sites, corresponding Carnegie stages, and major developmental observations. Samples were obtained between 2022 and 2023 from donors undergoing elective termination of pregnancy, following provision of written informed consent. Donors reported no known genetic or medical conditions. Exclusion criteria included fetal anomalies, abnormal growth (large or small for gestational age), exposure to chemical substances, and any self-reported donor medical conditions. Three samples, gestational week (GW) 18, were used in this study: a male and a female GW 18, and a male GW 18.4. Sex was determined using PCR. Fetal gestational age was assigned using ultrasound (ACUSON Juniper Juniper™ Ultrasound System; Siemens Healthineers, Ottawa, Ontario) by measuring the femur length, biparietal diameter, and foot length and then confirmed using a growth table (100). These gestational ages have been converted into FWs to align with existing data used for comparison (3). As such, GW 18 was included as FW 16, and GW 18.4 was included as FW 16.4. The time between collecting and receiving the tissue in our laboratory was less than 4 h. Samples were collected and dissected in ice-cold Hanks’ Balanced Salt Solution (HBSS) (Wisent, Montreal, Quebec; #311-512-CL) with 1% 1 M HEPES (Wisent, Montreal, Quebec; #330–050 EL). Spiral ganglia tissues were dissected from the intact cartilaginous otic capsule, sensory epithelium, and modiolus. Two samples (female FW 16.0 and male FW 16.4) were treated for 10 min at 37 °C in 2 mg/mL thermolysin from *Geobacillus stearothermophilus* (Millipore-Sigma, Oakville, Ontario; #P1512) to decrease the amount of surrounding mesenchyme and then dissected in the dissection solution containing fetal bovine serum (Thermo Fisher Scientific, Toronto, Ontario; #12484028) to immediately reduce the enzymatic activity, followed by washing steps with HBSS supplemented with 1% HEPES. Samples were placed in 1.5-mL DNA LoBind tubes (Eppendorf®, Mississauga, Ontario; #022431081) and then flash-frozen and stored in liquid nitrogen.

### Nuclei isolation and sequencing

The nuclei isolation protocol from 10x Genomics (Chromium Nuclei Isolation Kit, User Guide CG000505) was modified. Briefly, 500 μL of lysis buffer was added to the tube containing the sample, incubated on ice for 1 min, and mechanically triturated with a P1000 pipette for up to 9 min; cell lysis was assessed throughout this process. Following cell lysis, nuclei were passed through a 40 μm Flowmi^®^ Cell Strainer (Sigma-Aldrich, Oakville, Ontario) and then centrifuged at 500 rcf for 3 min, and the pellet was resuspended in Debris Removal Buffer and centrifuged at 700 rcf for 10 min. The pellet was then resuspended in wash buffer and centrifuged at 500 rcf for 10 min, and nuclei were resuspended in resuspension buffer. To increase nuclei quantity, two out of the three samples were processed following an optimized protocol whereby, after cell lysis, nuclei were washed in 500 μL wash buffer and resuspension buffer (1:1). Nuclei quality was assessed throughout the protocol, and intactness was over 90% on average. The resuspended nuclei were loaded into the Chromium Chip (full capacity well) and processed following the Chromium Next GEM Single Cell Multiome ATAC + Gene Expression workflow. cDNA libraries were sequenced using Illumina NovaSeq 6000 and NovaSeq X. Raw BCL Illumina files were converted to FASTQ files using the 10x Genomics Cell Ranger pipeline for demultiplexing and feature counting to generate gene expression matrices.

### Bioinformatics analysis: data preprocessing

Two HIE single-nucleus RNA-sequencing (snRNA-seq) datasets were analyzed in this study: one self-generated and another by van der Valk et al. (3), which focuses on characterizing inner ear sensory development during fetal stages. Both datasets were preprocessed independently using the same pipeline described below.

We initiated preprocessing with the filtered unique molecular identifier (UMI) count matrices provided by the original studies, which were generated using the 10x Genomics Cell Ranger pipeline. To ensure data quality, we applied an initial quality control (QC) step to remove low-quality nuclei, including those expressing more than 8,000 genes, as well as those with mitochondrial transcript content exceeding 5%, indicative of potential cellular stress or ambient RNA contamination. Despite this initial QC, the total UMI distribution suggested the presence of doublets. Therefore, we applied the doublet removal algorithm following cell clustering (will be described later) to mitigate this issue.

We then applied the Seurat pipeline to normalize gene expression using log normalization, with the median total UMI count as the scaling factor. Next, we identified the 2,000 most variable features (VFs) for each sample, scaled these VFs, and performed PCA (101). Subsequently, we applied Seurat’s canonical correlation analysis (CCA)-based integration on the PCA space to harmonize the datasets, generating a lower-dimensional representation that preserves biologically coherent cell identities shared across samples (102).

Cell clustering was performed using Seurat’s shared nearest neighbor (SNN)–Leiden approach on the integrated CCA space to identify major cell populations. Cell clusters were then manually annotated based on marker genes identified through DE analysis using the MAST framework (103). Finally, scDblFinder (109) was applied to detect and remove doublets within each sample, leveraging the identified cell clusters to refine doublet classification.

After QCs, the van der Valk et al. (3) dataset containing 23,792 cells was filtered down to 20,323 cells, and the self-generated dataset containing 30,838 cells was filtered to 21,369 cells for macrophage subset extraction. A final QC summary of total UMI counts, ribosomal content, and mitochondrial content in macrophages is provided in **Supplementary Figure 7**.

### Identify human inner ear macrophage subtypes

From each preprocessed dataset, the macrophage population underwent subset extraction based on the expression of the marker genes *PTPRC* (CD45) and *ITGAM* (CD11b) (**Supplementary Figure 1A2,B2**). The selected macrophages were then combined into a single dataset, comprising 48, 50, 353, 149, and 83 cells from FWs 7.5, 9.2, 16, 16.4, and adult samples, respectively, with median gene counts of 1,085, 2,395, 2,373, 1,826, and 2,459. Following the same integration pipeline as applied to the full dataset, the macrophage subset was reprocessed using Seurat, performing VF selection and PCA, followed by CCA integration on the PCA space, treating samples as individual batches. Uniform manifold approximation and projection (UMAP) was applied to the PCA and the CCA space to visualize macrophage populations before and after integration, respectively.

Macrophage subtypes were identified by performing SNN–Leiden clustering on the CCA space, setting the cluster resolution to 1, which resulted in seven clusters. This resolution was chosen to maximize the number of clusters while ensuring that each exhibited distinctly DEGs as revealed by MAST DE analysis (103). A gene was considered DE in a cluster if it was expressed in at least 25% of the cells within the cluster, exhibited a log-fold change greater than 0.5, and had an adjusted p-value less than 0.05.

To functionally profile macrophage subtypes, over-representation analysis (ORA) of gene ontology (GO) terms associated with biological pathways was performed for DEGs in each cluster using ClusterProfiler (104). GO terms with 10–500 genes were included, and those with an adjusted p-value below 0.05 were considered enriched. To remove redundancy and retain the most representative terms, the simplify function in ClusterProfiler was applied to reduce redundancy in enrichment results.

### Profile inner ear macrophage identity by module gene expression

To characterize macrophage identity, we analyzed the expression of a predefined set of macrophage module genes, as identified by Wang et al. (46). We derived these marker genes from a comprehensive immune atlas of human fetal development, spanning multiple tissue types, and used them to classify primary macrophage subtypes. To explore how IEMs of different subtypes and ages express the module genes, we performed two key analyses: first, we conducted PCA on the expression of the module genes, generating a PCA space that is segregated by distinct modules. We projected macrophages onto this PCA space and visualized them alongside gene loadings using a PCA biplot. Second, we quantified the relative expression level of each module in individual cells using AUCell (47), summarizing cellular identity as a vector of identity scores for each gene module.

### Trace inner ear macrophage lineage by projecting onto an integrated myeloid atlas

To trace macrophage lineage, we queried our data against an integrated bulk gene expression atlas of myeloid cells. This atlas comprises samples from 44 independent studies, encompassing myeloid biology across diverse culture environments and various developmental stages. Here, “querying” refers to projecting the query macrophage data onto the PCA space of the atlas. To achieve this, we applied the Sincast framework (48) to impute the single-cell query and align its distribution with the bulk atlas, enabling meaningful projection. The PCA of the atlas was performed on the 1,922 genes shared between the atlas and the top 15,000 VFs of the query. The projection aligns the query cells with a stable gene expression space established by the atlas, highlighting biological differences while reducing technical noise. Therefore, leveraging the mapped query PC scores, we applied Slingshot (57) to make a robust estimation of pseudotime and cell lineage, with Mac 1 as the starting point for differentiation.

### Reveal differences in inner ear macrophage signaling interactions during fetal development

We investigated macrophage signaling interaction unique to three age groups: early fetal weeks (FWs 7.5 and 9.2), middle fetal weeks (FWs 16 and 16.4), and adult (one sample). First, we performed DE analysis to pinpoint genes upregulated in each group. Next, we used curated prior knowledge databases to infer potential intra- and intercellular signaling interactions associated with these DEGs. It is important to note that FW 7.5 and 9.2 represent two distinct developmental stages. Ideally, they should be analyzed separately rather than grouped under a single “early fetal week” category. However, because DE analysis was performed at the pseudobulk level (see below), each age corresponds to a single sample. Conducting DE analysis between a single-sample group and others would be statistically unreliable, even though theoretically possible. Therefore, we grouped FW 7.5 and 9.2 together to increase statistical robustness.

For DE, we aggregated macrophages from each sample into pseudobulk profiles and rank-normalized their expression to reduce technical variability that may confound the analysis (105). We performed DE to compare each age group with the two others using the limma pipeline, chosen for its suitability with limited sample sizes (106). For each gene examined in a given age group, we calculated a *π*-value by multiplying the gene’s fold change by the negative log10 of its p-value. We used the *π*-value to determine the extent of DE (107). To functionally comprehend age differences, we performed GSEA on the resulting *π*-value-ordered list, focusing on GO biological pathway terms. Using ClusterProfiler, we tuned and refined GSEA as described for ORA on DEGs of macrophage subtypes.

To infer TF activity representing intracellular gene regulation, we applied the DecoupleR algorithm (61) to each group’s *π*-values. This approach estimates TF activity by measuring the correlation between the TF’s known regulatory interactions (sourced from the CollectTRI database) and the observed *π*-values of target genes (108).

To extend beyond individual TF inference and elucidate overarching signaling dynamics across developmental stages, we employed NeighbourNet analysis (78) to reconstruct GRNs for age-specific marker genes (top 50 upregulated genes per age group based on *π*-values) and their potential upstream signaling pathways. For the markers of a given age group, NeighbourNet constructs GRNs at the level of individual cells and subsequently clusters and aggregates these networks to represent the principal gene regulatory patterns shared among cells. The top two aggregated GRNs for each of the early and middle FW markers are displayed in **Figure 4**. Finally, we utilized NicheNet to investigate intercellular signaling interactions, specifically ligand–receptor binding events implicated in gene upregulation within each age group (79). For this analysis, we provided the identified age markers as target inputs. Then, NicheNet predicted upstream ligands and their corresponding receptors and inferred their regulatory effects on the provided target genes.

## Supplementary results

### NicheNet prioritization of ligands that target age-specific inner ear macrophage markers

We used NicheNet (79) to explore intercellular signaling between macrophages and other inner ear cell types during development. Specifically, we examined receptor–ligand interactions predicted to drive the upregulation of age markers derived from early FWs (**Supplementary Figure 6A**). Early FW IEMs showed enriched signaling with chondrocytes (*CD74*-*COPA* and *NRP1*-*SEMA3C/3D*), cochlear epithelium (*TGFBR1/2/3*-*TGFB2* and *FZD2/6*-*SFRP1*), melanocytes (*TGFBR1/2/3*-*TGFB2* and *GJB2*-*GJB6*), neurons (*FGFR1/2*-*FGF10* and *NRP1/2*-*SEMA3E*), and IEMs themselves (*PDGFRA/B*, *PLXNA1*-*NRP1*, and *TGFBR1/2/3*-*TGFB1*). These interactions highlight an early activation of growth factor pathways *TGFB1/2* and *FGF1/10*, which are predicted to upregulate genes such as *HMOX1*, *TOP2A*, *SEMA3A/3C*, *HMGA2*, and *ADAMTS19*. Collectively, these results support a previously unidentified trophic role for early IEMs during development (**Figure 1D**). Notably, some of the TGF-β and FGF targets are also implicated in neural outgrowth and pathfinding.

A similar NicheNet analysis was applied to age markers derived from middle FWs (**Supplementary Figure 6B**). In comparison, the middle FW IEMs were predicted to adopt a classical macrophage phenotype and predominantly communicate with mesenchymal, endothelial, and other IEMs. It should be reiterated that the tissues collected at this donor age were isolated exclusively from the cochlear modiolus. Therefore, these data should be interpreted within the context of this specific inner ear location. As such, the cell populations shown in **Supplementary Figure 1A1** were enriched; however, cochlear epithelial cells (including hair and supporting cells), as well as cells of the stria vascularis and lateral cochlear wall, were absent. Our analyses reveal that modiolar macrophages were predicted to communicate with ACAN + mesenchymal stem cells (*IGSF11*-*IGSF*), endothelial cells (*TNFSFRSF10A/B/C/D/11B*-*TNFSF10*, *ERBB2*-*HLA-A*, *LILRB1/2*-*HLA-A*, and *KLRB1/KLRF1*-*CLEC2B*), and modiolar macrophages themselves (*CD4/9/37/53/63/81/82*-*HLA-DRA*). Collectively, these predicted signaling interactions suggest age-dependent regulation mediated by *VEGFA* and *TNFSF10* family ligands, targeting genes essential for core macrophage functions, including phagocytosis (*CDKN1A*), wound healing (*HBEGF* and *PLAU*), and efferocytosis (*ANXA1*). Interestingly, the predicted target genes also spanned both known inflammatory (*BHLHE40*, *PPP1R15A*, *PTGS2*, *IL2RA*, and *SOCS3*) and reparative (*CXCL8*, *DUSP1*, *KLF2*, *KLF4*, *MAFF*, *PLAU*, *ATF3*, *SOCS3*, *PPIF*, *CITED2*, *FOSL2*, *NFIL3*, and *RGS2*) immune response programs, reflecting a possible surveillant or transitional activation state in modiolar macrophages at this stage.

## Code availability

The processed macrophage subset of the data, along with the code for reproducing all the bioinformatics analyses conducted, has been deposited in the Zenodo repository (https://zenodo.org/records/15328483).

## Data Availability

The original contributions presented in the study are publicly available. This data can be found here: doi.org/10.5281/zenodo.15328483 and https://github.com/meiosis97/Inner-ear-macrophage/tree/main.

## Supplementary material

Supplementary Figure 1 Selection of macrophage subsets from human inner ear snRNA-seq data. **(A)** UMAP plots showing the distribution of cell types (deposited in https://zenodo.org/records/15328483). A2: Nebulosa highlights the density of expression of two macrophage markers used for macrophage selection: PTPRC (CD45) and ITGAM (CD11b). **(B)** Similar to **(A)**, but showing the UMAP plots of (3). **(C)** 𝜋-value (y-axis: negative log p-value × log fold change) indicates the level of differential expression of DEGs, identified for each macrophage subtype (x-axis). **(D)** Correlation matrix displaying the relationship between different macrophage subtypes’ pseudobulk expression profiles in DEGs.

Supplementary Figure 2 Macrophage marker genes. Expression of common macrophage markers, and top ranked discriminating genes (first 7 genes) in each subtype. In addition to showing the expression in each age group independently to demonstrate the consistency of markers across age groups, the figure notation follows the same style as **Figure 1**.

Supplementary Figure 3 Growth factor signaling Interpreted as in **Figure 1D**, this figure profiles growth−factor and receptor expression related to NRG and WNT signaling across inner−ear cell types. **(A)** Growth−factor (ligand) expression in macrophages. **(B)** and **(C)** WNT and NRG receptor expression in early fetal week (Week 7.5 and 9.2) and middle fetal week (week 16 and 16.4) samples, respectively.

Supplementary Figure 4 Benchmarking inner ear macrophage identity using Data from (13). **(A)** UMAP embedding of the (13) dataset, based on the 2,000 most variable features. Cells are colored by their assigned cell type, tissue of origin, and developmental stage. **(B)** Expression of the first five celltype markers. Cell types are shown on the y-axis, and gene symbols on the x-axis. The figure notation follows the same style as **Figure 1**. **(C)** Projection of query IEMs onto the UMAP in **(A)**. Panels (C1) and (C2) show query cells colored by age and by macrophage subtype, respectively. **(D)** UMAP embedding of the (13) dataset based on upregulated tissue-specific markers. **(E)** Similar to **(B)**, but showing expression of the first five tissue-specific markers. **(F)** Similar to **(C)**, with the query IEMs projected onto the UMAP in (D).

Supplementary Figure 5 Extended trajectory analysis **(A–C)** are similar to **Figure 2****(F–H)**, but pseudotime is recalculated in Slingshot using fetal week (FW) 7.5 macrophages as the root. **(D)** Mirrors **Figure 2****(C)**, but showing the projecting the fetal macrophage data from (13) onto the reference myeloid atlas. **(E)** Side−by−side comparison of Bian’s projection (L) and the IEM projection (R) reveals that early inner ear macrophages (FWs 7.5 and 9.2) overlap with the yolk−sac macrophage lineage present in the Bian’s study.

Supplementary Figure 6 Prioritizing ligands with high regulatory potential on the top 50 age markers using NicheNet. Age markers are the upregulated genes of A age group derived from the differential gene expression analysis in **Figure 3A**. **(A)** NicheNet ligand prioritization using the age markers of early fetal weeks (FWs 7.5 and 9.2) inner ear macrophages (IEMs) as the targets of interests. **(B)** Similar to **(A)**, but showing NicheNet analysis on the age markers of middle FWs (FWs 16 and 16.4) IEMs. Pink heatmap: NicheNet ligand (row) - receptor (column) interaction weights. Purple heatmap: NicheNet ligands (row) on targets (column) regulatory potential. Aligned to the rows of the heatmaps is the dotplot showing the expression of the prioritized ligands across inner ear cell types. Aligned to the columns of the heatmaps are the dotplots showing the expression of predicted receptors and targets within IEMs.

Supplementary Figure 7 Quality checking of inner ear macrophages Quality checking of IEMs by assessing ribosomal content, mitochondrial content, and total UMI counts. Macrophages are grouped by **(A)** subtype and **(B)** age. No strong association is observed between any QC metric and macrophage grouping, suggesting overall high data quality after pre-processing.
