# Supplementary material for: Trophic and temporal dynamics of macrophage biology in human inner ear organogenesis
Source: Front Immunol. 2025 Nov 18;16:1690583. doi: 10.3389/fimmu.2025.1690583 (PMC12669117; doi:10.3389/fimmu.2025.1690583)
Supplement: Supplementary Figure 1 — Selection of macrophage subsets from human inner ear snRNA-seq data. (A) UMAP plots showing the distribution of cell types (deposited in https://zenodo.org/records/15328483). A2: Nebulosa highlights the density of expression of two macrophage markers used for macrophage selection: PTPRC (CD45) and ITGAM (CD11b). (B) Similar to (A), but showing the UMAP plots of (3). (C) 𝜋-value (y-axis: negative log p-value × log fold change) indicates the level of differential expression of DEGs, identified for each macrophage subtype (x-axis). (D) Correlation matrix displaying the relationship between different macrophage subtypes’ pseudobulk expression profiles in DEGs. [file DataSheet1.docx]

# Supplementary Results

## NicheNet prioritization of ligands that target age-specific inner ear macrophage markers

We used NicheNet ([79](#ref-browaeys2020nichenet)) to explore intercellular signaling between macrophages and other inner ear cell types during development. Specifically, we examined receptor-ligand interactions predicted to drive the upregulation of age markers derived from early FWs (Supplementary Figure [6](#suppfigure6)A). Early FWs IEMs showed enriched signaling with chondrocytes (*CD74*-*COPA* and *NRP1*-*SEMA3C/3D*), cochlear epithelium (*TGFBR1/2/3*-*TGFB2* and *FZD2/6*-*SFRP1*), melanocytes (*TGFBR1/2/3*-*TGFB2* and *GJB2*-*GJB6*), neurons (*FGFR1/2*-*FGF10* and *NRP1/2*-*SEMA3E*), and IEMs themselves (*PDGFRA/B*, *PLXNA1*-*NRP1*, and *TGFBR1/2/3*-*TGFB1*). These interactions highlight an early activation of growth factor pathways *TGFB1/2* and *FGF1/10*, which are predicted to upregulate genes such as *HMOX1*, *TOP2A*, *SEMA3A/3C*, *HMGA2*, and *ADAMTS19*. Collectively, these results support a previously unidentified trophic role for early IEMs during development (Figure [1](#figure1)D). Notably, some of the TGF-$\beta$ and FGF targets are also implicated in neural outgrowth and pathfinding.

A similar NicheNet analysis was applied to age markers derived from middle FWs (Supplementary Figure [6](#suppfigure6)B). In comparison, the middle FWs IEMs were predicted to adopt a classical macrophage phenotype and predominantly communicate with mesenchymal, endothelial, and other IEMs. It should be reiterated that the tissues collected at this donor age were isolated exclusively from the cochlear modiolus. Therefore, these data should be interpreted within the context of this specific inner ear location. As such, the cell populations shown in Supplementary Figure [1](#suppfigure1)A1 were enriched; however, cochlear epithelial cells (including hair and supporting cells), as well as cells of the stria vascularis and lateral cochlear wall, were absent. Our analyses reveal that modiolar macrophages were predicted to communicate with ACAN + mesenchymal stem cells (*IGSF11*-*IGSF*), endothelial cells (*TNFSFRSF10A/B/C/D/11B*-*TNFSF10*, *ERBB2*-*HLA-A*, *LILRB1/2*-*HLA-A*, and *KLRB1/KLRF1*-*CLEC2B*), and modiolar macrophages themselves (*CD4/9/37/53/63/81/82*-*HLA-DRA*). Collectively, these predicted signaling interactions suggest age-dependent regulation mediated by *VEGFA* and *TNFSF10* family ligands, targeting genes essential for core macrophage functions, including phagocytosis (*CDKN1A*), wound healing (*HBEGF* and *PLAU*), and efferocytosis (*ANXA1*). Interestingly, the predicted target genes also spanned both known inflammatory (*BHLHE40*, *PPP1R15A*, *PTGS2*, *IL2RA*, and *SOCS3*) and reparative (*CXCL8*, *DUSP1*, *KLF2*, *KLF4*, *MAFF*, *PLAU*, *ATF3*, *SOCS3*, *PPIF*, *CITED2*, *FOSL2*, *NFIL3*, and *RGS2*) immune response programs, reflecting a possible surveillant or transitional activation state in modiolar macrophages at this stage.

# Supplementary Figures


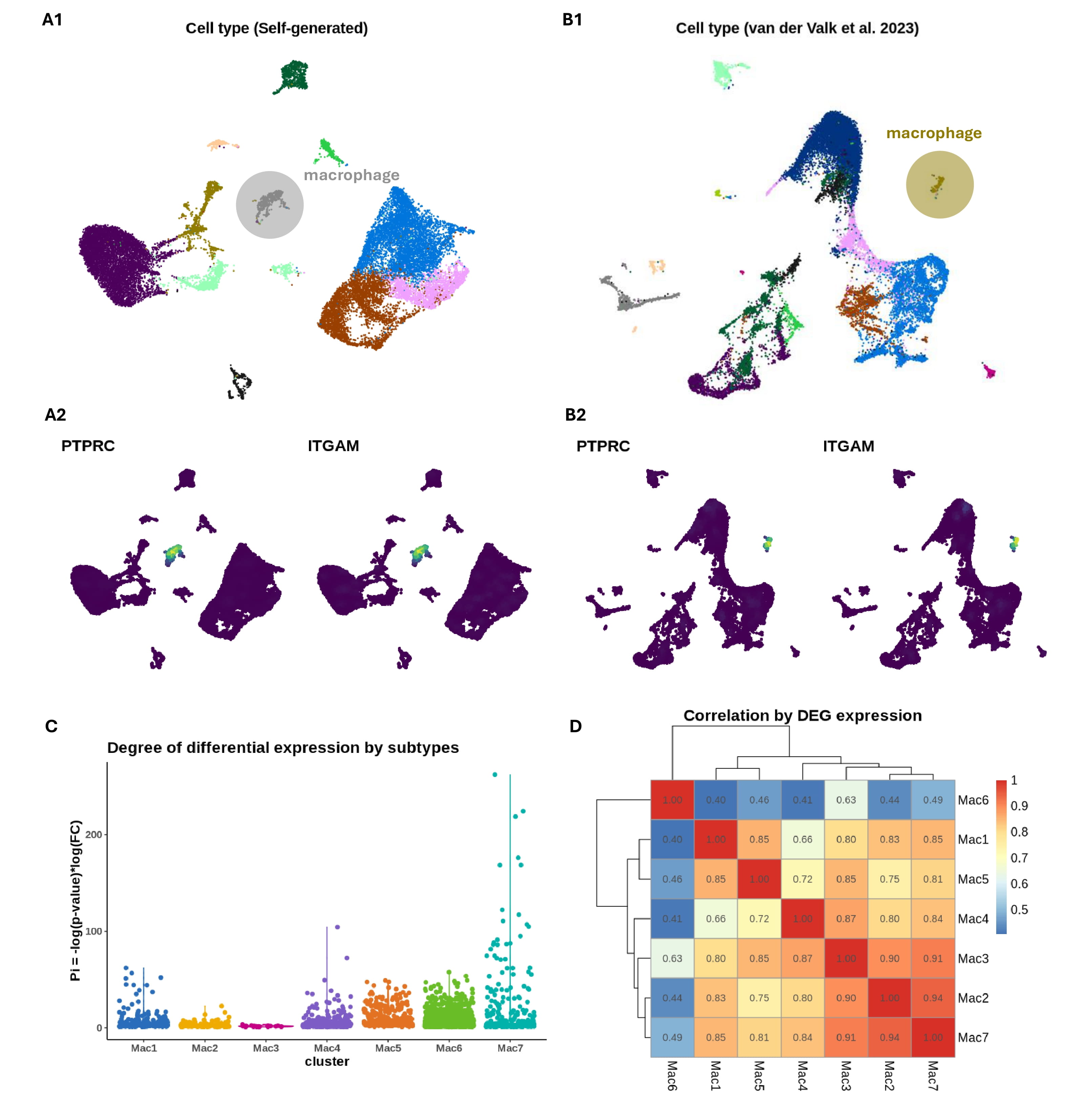


**Supplementary Figure 1. Selection of macrophage subsets from human inner ear snRNA-seq data.** **(A)** UMAP plots showing the distribution of cell types (deposited in <https://zenodo.org/records/15328483>). A2: Nebulosa highlights the density of expression of two macrophage markers used for macrophage selection: PTPRC (CD45) and ITGAM (CD11b). **(B)** Similar to (A), but showing the UMAP plots of ([3](#ref-van2023single)). **(C)** $\pi$-value (y-axis: negative log p-value × log fold change) indicates the level of differential expression of DEGs, identified for each macrophage subtype (x-axis). **(D)** Correlation matrix displaying the relationship between different macrophage subtypes’ pseudobulk expression profiles in DEGs.


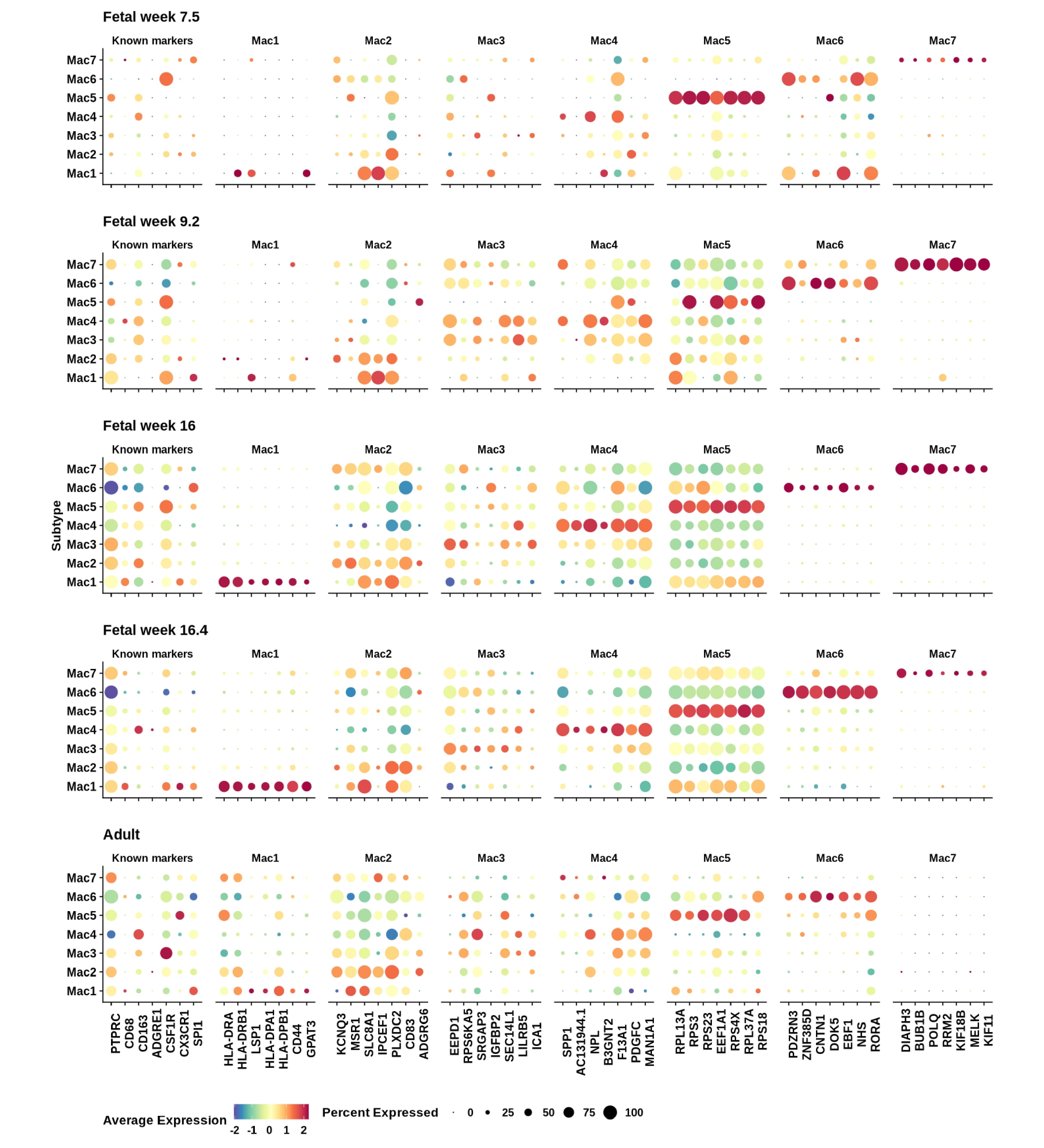


**Supplementary Figure 2. Macrophage marker genes**. Expression of common macrophage markers, and top ranked discriminating genes (first 7 genes) in each subtype. In addition to showing the expression in each age group independently to demonstrate the consistency of markers across age groups, the figure notation follows the same style as Figure [1](#figure1).


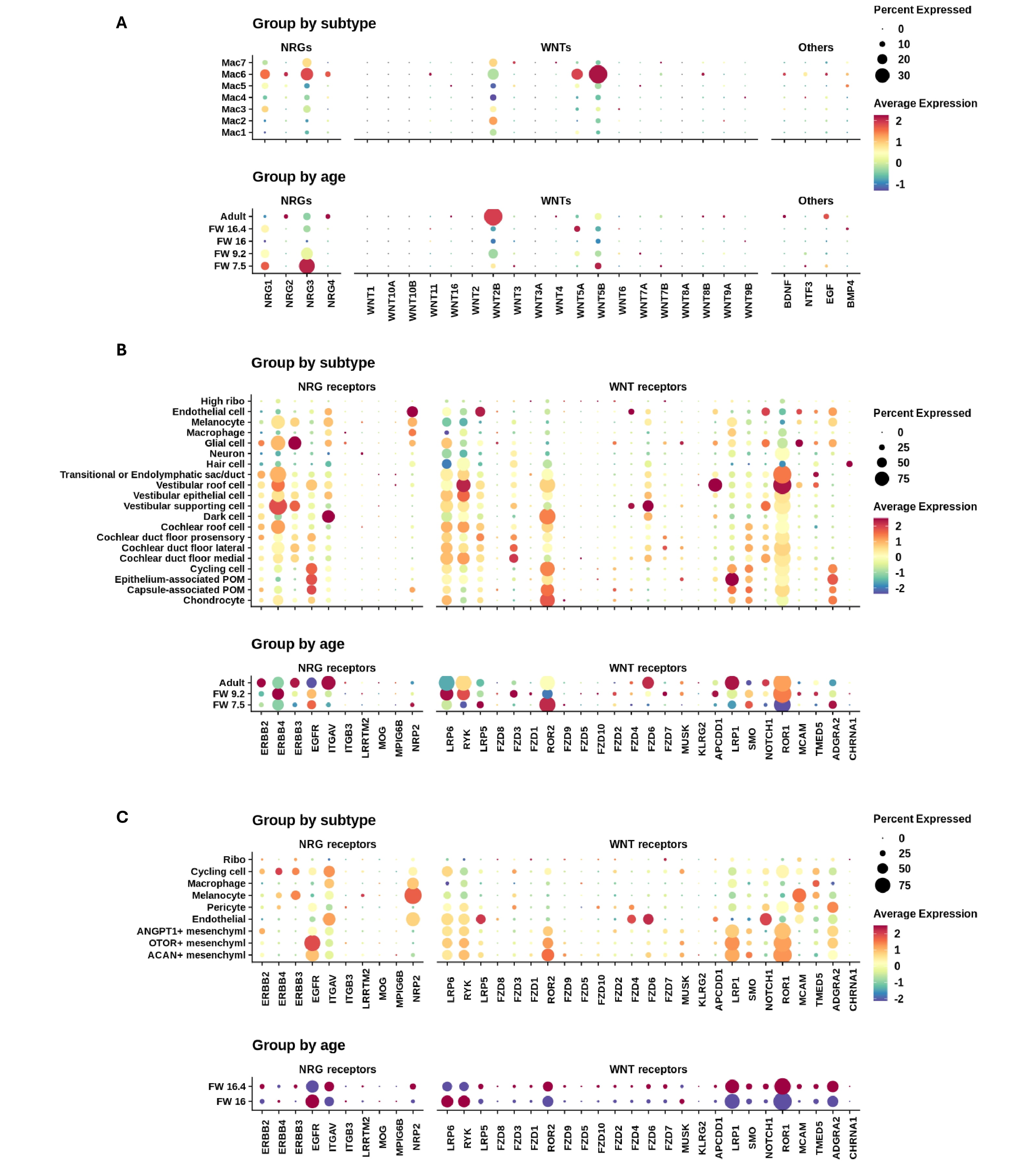


**Supplementary Figure 3. Growth factor signaling** Interpreted as in Figure [1](#figure1)D, this figure profiles growth‑factor and receptor expression related to NRG and WNT signaling across inner‑ear cell types. **(A)** Growth‑factor (ligand) expression in macrophages. **(B)** and **(C)** WNT and NRG receptor expression in early fetal week (Week 7.5 and 9.2) and middle fetal week (week 16 and 16.4) samples, respectively.


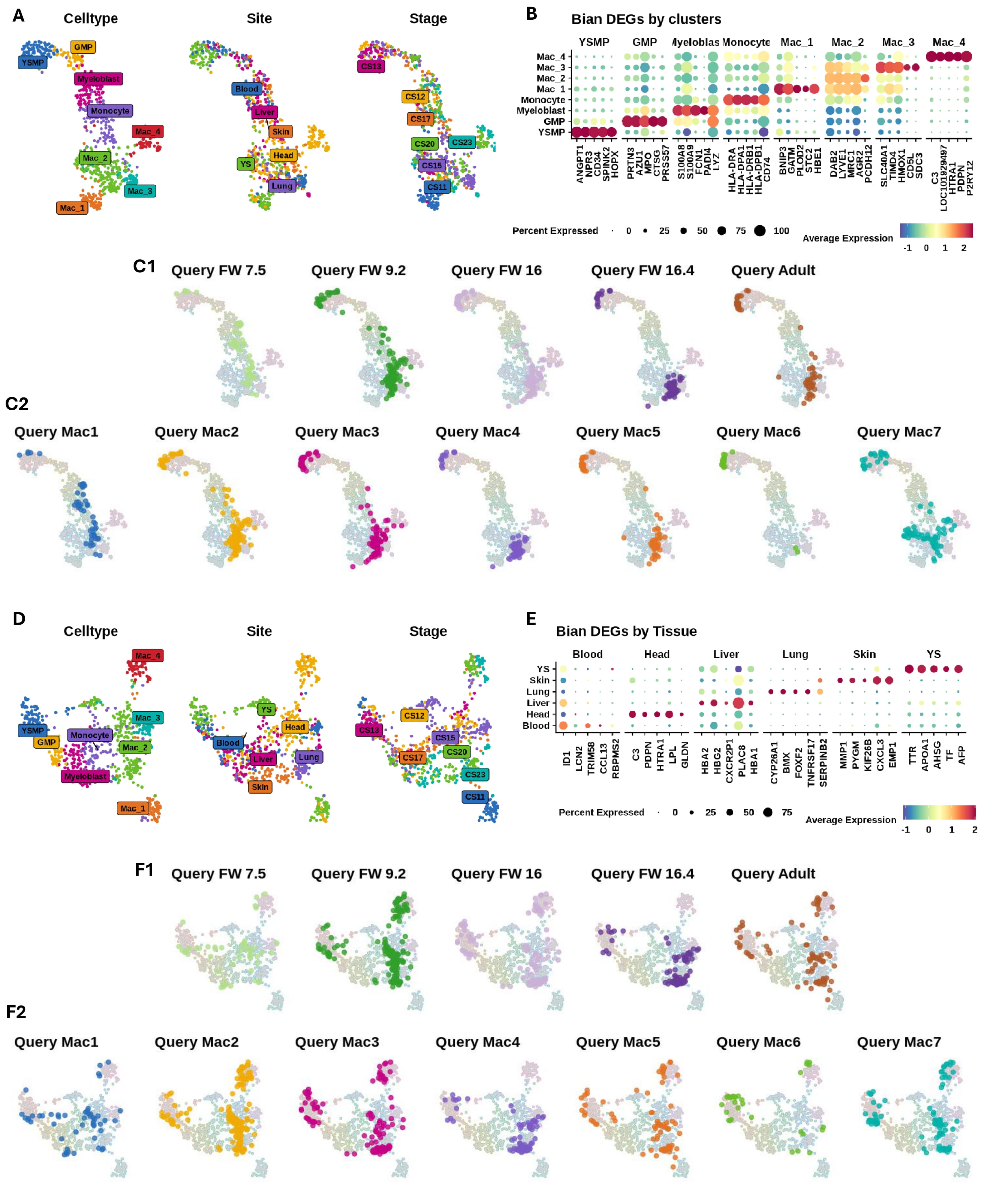


**Supplementary Figure 4. Benchmarking inner ear macrophage identity using Data from (**[**13**](#ref-bian2020deciphering)**).** **(A)** UMAP embedding of the ([13](#ref-bian2020deciphering)) dataset, based on the 2,000 most variable features. Cells are colored by their assigned cell type, tissue of origin, and developmental stage. **(B)** Expression of the first five celltype markers. Celltypes are shown on the y-axis, and gene symbols on the x-axis. The figure notation follows the same style as Figure [1](#figure1). **(C)** Projection of query IEMs onto the UMAP in (A). Panels (C1) and (C2) show query cells colored by age and by macrophage subtype, respectively. **(D)** UMAP embedding of the ([13](#ref-bian2020deciphering)) dataset based on upregulated tissue-specific markers. **(E)** Similar to (B), but showing expression of the first five tissue-specific markers. **(F)** Similar to (C), with the query IEMs projected onto the UMAP in (D).


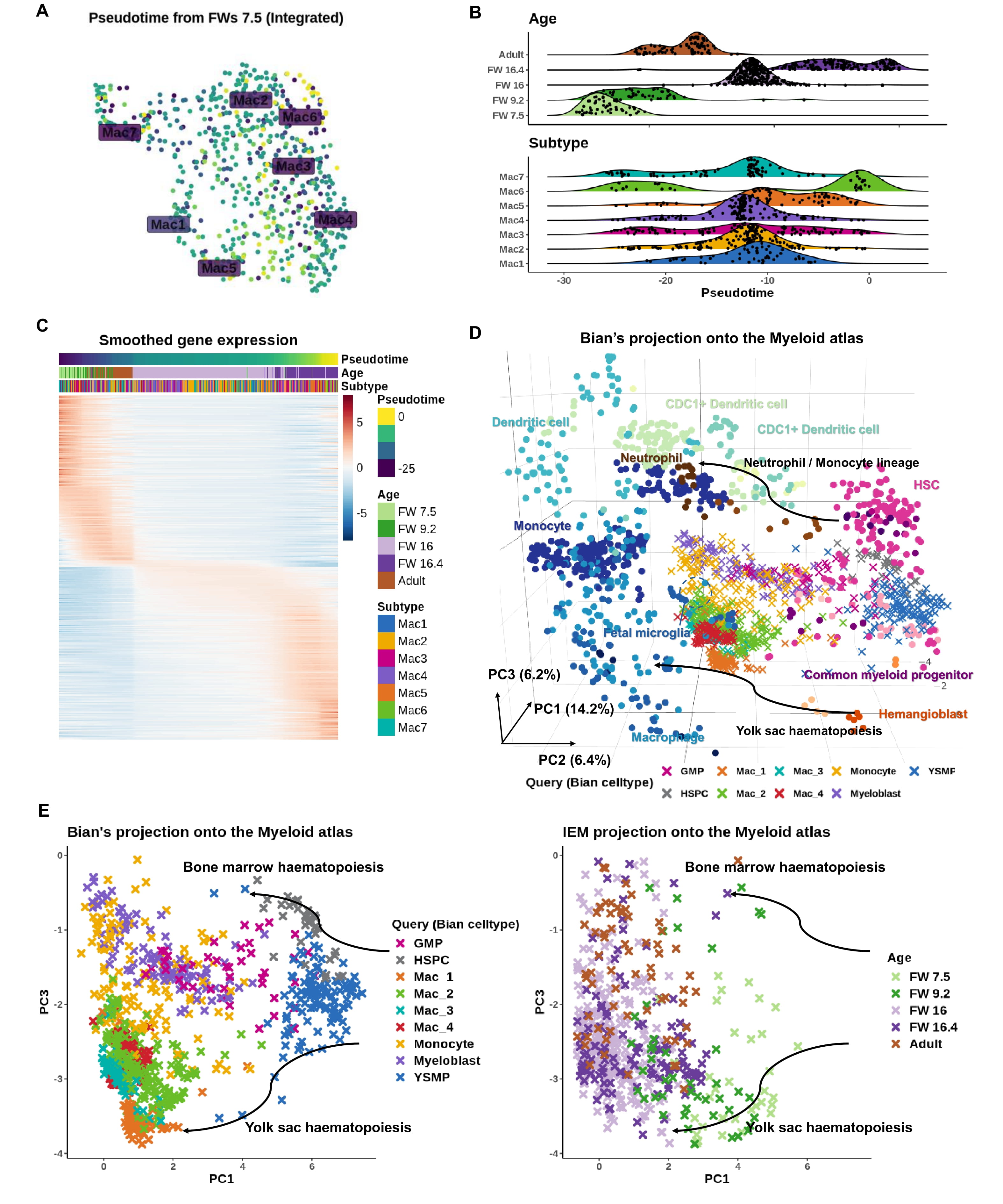


**Supplementary Figure 5. Extended trajectory analysis** (A-C) are similar to Figure [2](#figure2)(F-H), but pseudotime is recalculated in Slingshot using fetal week (FW) 7.5 macrophages as the root. **(D)** Mirrors figure [2](#figure2)(C), but showing the projecting the fetal macrophage data from ([13](#ref-bian2020deciphering)) onto the reference myeloid atlas. **(E)** Side‑by‑side comparison of Bian’s projection (L) and the IEM projection (R) reveals that early inner ear macrophages (FWs 7.5 and 9.2) overlap with the yolk‑sac macrophage lineage present in the Bian’s study.


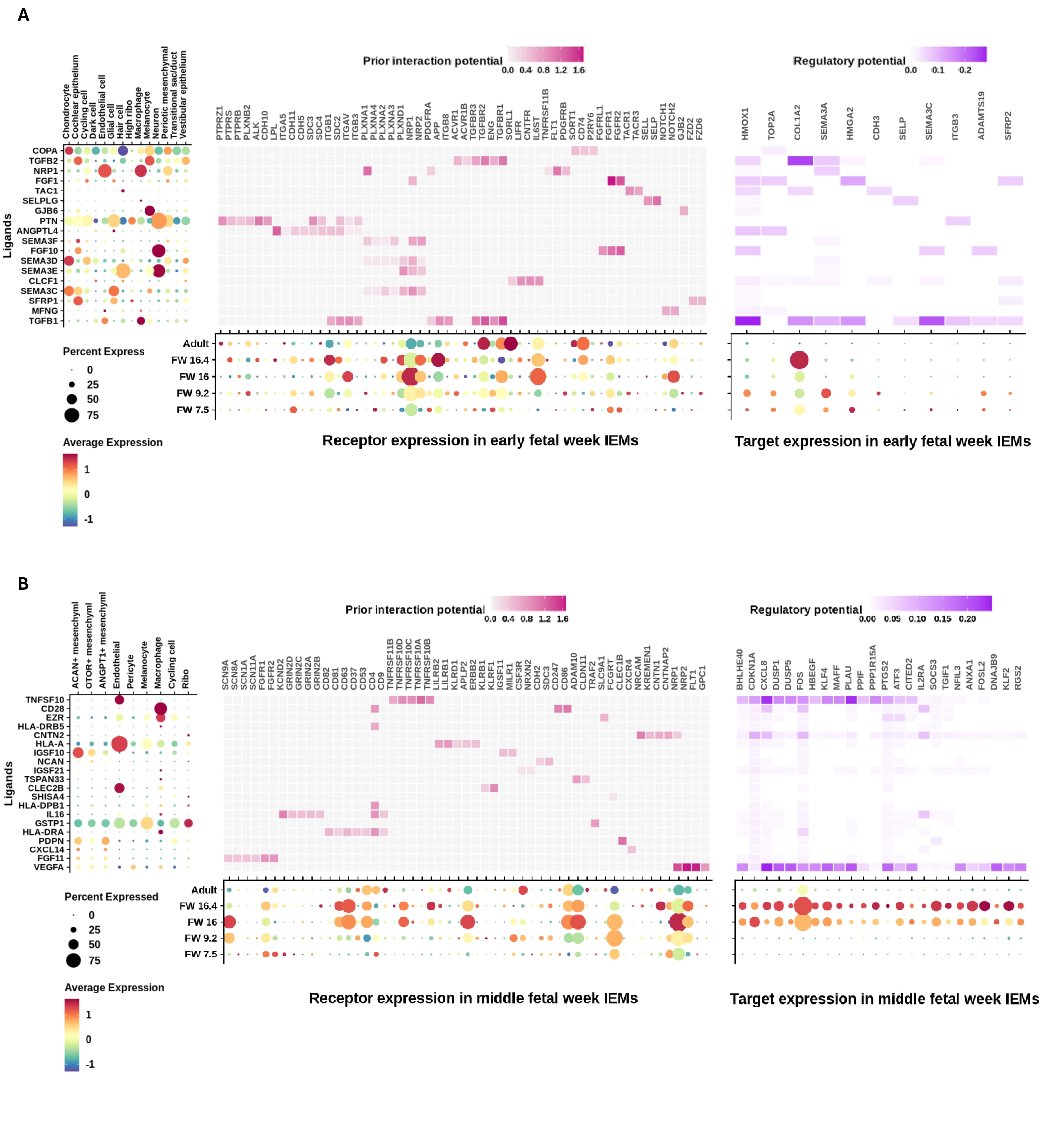


**Supplementary Figure 6. Prioritizing ligands with high regulatory potential on the top 50 age markers using NicheNet**. Age markers are the upregulated genes of A age group derived from the differential gene expression analysis in Figure [3](#figure3)A. **(A)** NicheNet ligand prioritization using the age markers of early fetal weeks (FWs 7.5 and 9.2) inner ear macrophages (IEMs) as the targets of interests. **(B)** Similar to (A), but showing NicheNet analysis on the age markers of middle FWs (FWs 16 and 16.4) IEMs. Pink heatmap: NicheNet ligand (row) - receptor (column) interaction weights. Purple heatmap: NicheNet ligands (row) on targets (column) regulatory potential. Aligned to the rows of the heatmaps is the dotplot showing the expression of the prioritized ligands across inner ear cell types. Aligned to the columns of the heatmaps are the dotplots showing the expression of predicted receptors and targets within IEMs.


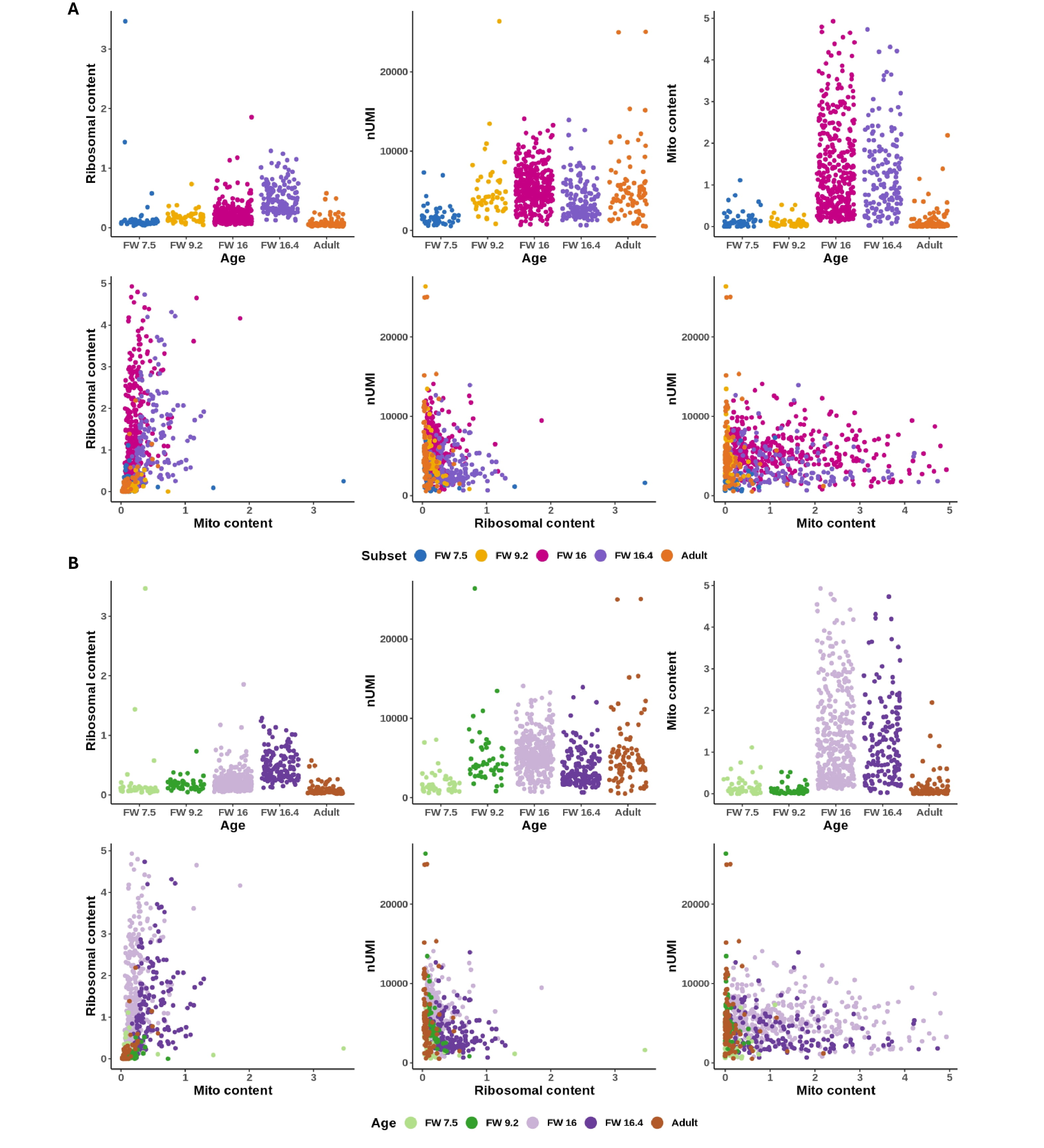


**Supplementary Figure 7. Quality checking of inner ear macrophages** Quality checking of IEMs by assessing ribosomal content, mitochondrial content, and total UMI counts. Macrophages are grouped by (A) subtype and (B) age. No strong association is observed between any QC metric and macrophage grouping, suggesting overall high data quality after pre-processing.
